# Hysteresis in Transport Critical-Current Measurements of Oxide Superconductors

**DOI:** 10.6028/jres.106.031

**Published:** 2001-08-01

**Authors:** L. F. Goodrich, T. C. Stauffer

**Affiliations:** National Institute of Standards and Technology, Boulder, CO 80305, USA

**Keywords:** magnetic field angle, critical current, high temperature superconductors, interlaboratory comparison, magnetic hysteresis, standard method, variable temperature

## Abstract

We have investigated magnetic hysteresis in transport critical-current (*I*_c_) measurements of Ag-matrix (Bi,Pb)_2_Sr_2_Ca_2_Cu_3_O_10–_*_x_* (Bi-2223) and AgMg-matrix Bi_2_Sr_2_CaCu_2_O_8+_*_x_* (Bi-2212) tapes. The effect of magnetic hysteresis on the measured critical current of high temperature superconductors is a very important consideration for every measurement procedure that involves more than one sweep of magnetic field, changes in field angle, or changes in temperature at a given field. The existence of this hysteresis is well known; however, the implications for a measurement standard or interlaboratory comparisons are often ignored and the measurements are often made in the most expedient way. A key finding is that *I*_c_ at a given angle, determined by sweeping the angles in a given magnetic field, can be 17 % different from the *I*_c_ determined after the angle was fixed in zero field and the magnet then ramped to the given field. Which value is *correct* is addressed in the context that the proper sequence of measurement conditions reflects the application conditions. The hysteresis in angle-sweep and temperature-sweep data is related to the hysteresis observed when the field is swept up and down at constant angle and temperature. The necessity of heating a specimen to near its transition temperature to reset it to an initial state between measurements at different angles and temperatures is discussed.

## 1. Introduction

**Fig. 1:**
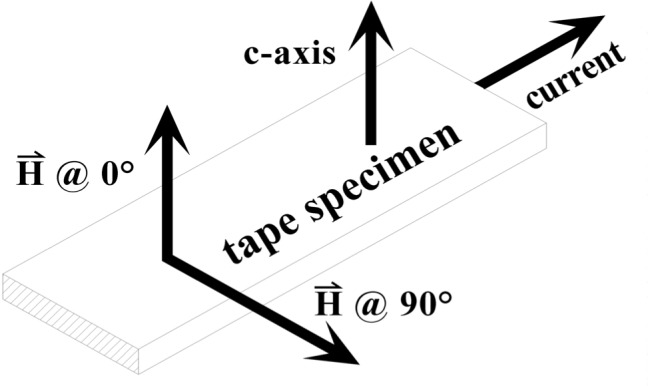
Illustration of the definition of the magnetic field angle with respect to the tape-specimen geometry and current.

Three quantities that are common variables in transport critical-current (*I_c_*) measurements on superconductors are temperature, magnetic field, and angle of the magnetic field. In this paper, the term temperature refers to the thermodynamic temperature, *T*, of the specimen in units of kelvin, K. The term magnetic field (or field) refers to the external applied magnetic-field strength, *H.* For convenience and consistency with the practice of the superconductor industry, we express our magnetic field in terms of *µ*_0_*H* in units of teslas, T, where *µ*_0_ = 4*π* × 10^–7^ H/m, the permeability of free space. For a tape specimen, the term angle, *θ*, refers to the angle between the magnetic field strength vector and the surface normal vector of the tape specimen (see Fig. 1). The *c*-axis of the textured superconductor tapes measured for this paper is perpendicular to the wide face of the tape. The field angle is defined as 0° when the field is parallel to the *c*-axis and as 90° when the field is parallel to the wide face of the tape. In this paper, the applied magnetic field is always perpendicular to the specimen current direction (see Fig. 1).

**Fig. 2:**
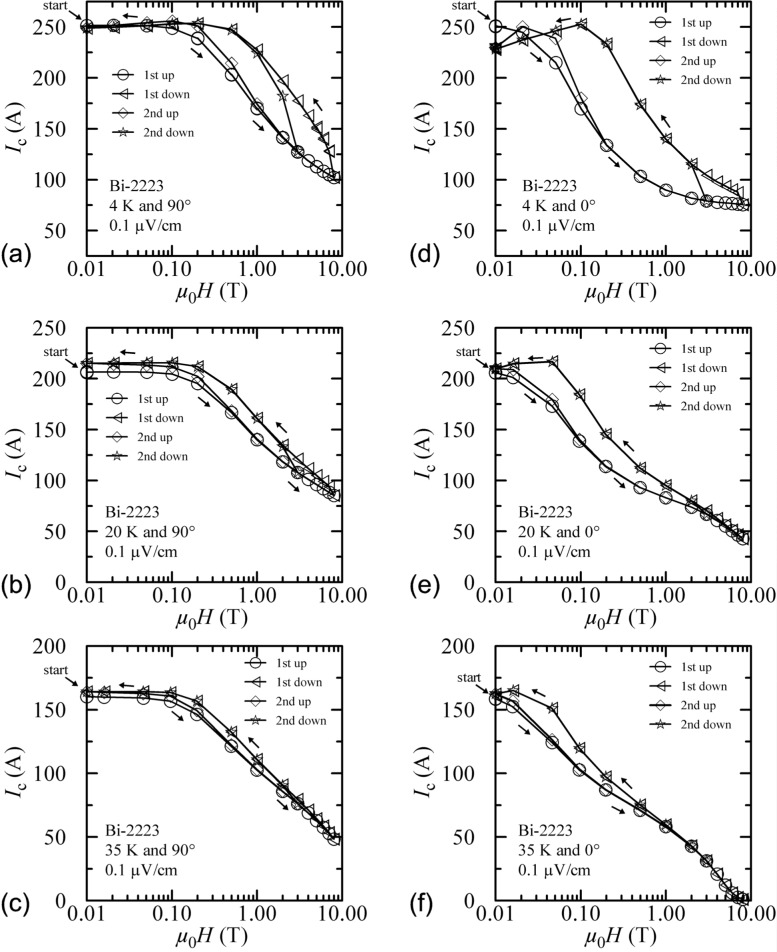
*I*_c_ at 0.1 µV/cm versus magnetic field for the Bi-2223 specimen for various field-sweep directions, temperatures, and angles. The order in the legend is the order that these data were taken and the arrows indicate the field-sweep direction: (a) 4 K and 90°, (b) 20 K and 90°, (c) 35 K and 90°, (d) 4 K and 0°, (e) 20 K and 0°, (f) 35 K and 0°.

Many papers [1]–[7] have reported magnetic hysteresis observed in transport critical-current measurements of oxide high-temperature superconductors (HTS). The most common observation of hysteresis is that the measured *I_c_* as a function of *H* is different when measured with increasing and decreasing field. Thus, *I_c_(H)* is a multi-valued function, as shown in Fig. 2. The measured *I_c_* of an HTS can depend significantly on its history of temperature and applied magnetic-field strength and angle. This phenomenon is referred to as magnetic hysteresis or *I_c_* hysteresis. The effect of *I_c_* hysteresis on the measured *I_c_* can be reset to an initial virgin state by heating the superconductor above its critical temperature *T_c_.* Thus, *I_c_*(*T, H, θ*) also depends on the history of these parameters.

This leads to the question, Which values are *“correct”*? The *correct* value is determined by the sequence of conditions the conductor experiences in the intended application. In many applications, such as a superconducting solenoid magnet, the conductor is cooled to some temperature in zero field. Then the magnetic field is increased to some maximum value, and its angle with respect to any portion of the conductor remains nearly constant. Of course the angle will be different for different portions of the magnet. In this application, any enhanced *I_c_* caused by the portion of the conductor being exposed to a previous higher field, a higher temperature, or other field angles is not relevant. Thus, the initial virgin values are *correct* since they are obtained after zero-field cooling from a temperature above *T_c_* and settling of the angle and temperature. In this paper, the terms “virgin” or *“correct”* identify data taken only after this specific sequence of conditions. This paper focuses on (a) the observed *I_c_* hysteresis, (b) what is the *correct* value of *I_c_*, and (c) what more practically expedient sequence of measurement conditions gives values that are closest to *correct.*

The measurement considerations in hysteresis-loss (ac-loss) measurements are different from those in critical-current measurements. The terminology sometimes used in hysteresis-loss measurements is: the first field sweep up is called the “initial branch,” the field sweep down is the “descending branch,” and the subsequent field sweep up is the “ascending branch.” In loss measurements, the worst case is a higher value, in contrast to *I_c_* measurements where the lowest value is the worst case. So the initial branch of the ac-loss hysteresis loop is discarded because the subsequent ascending branch results in a higher loss for the loop and the initial branch is not re-established when the field is cycled. All subsequent descending and ascending branches are the same, so there is a tendency to generalize that the repeatable part of a hysteretic property is the *correct* answer. This is true for hysteresis-loss measurements, but it is not generally *correct* for *I_c_* measurements that have hysteresis.

*I_c_* measurements over a wide range of many parameters (temperature, field, and angle) are needed given the broad scope of operating conditions in a variety of HTS applications. This characterization is very time consuming, especially if the specimen has to be heated above its *T_c_* and cooled in zero field after each field sweep and between each angle setting. This paper focuses on the observed *I_c_* hysteresis, what is the *correct* value of *I_c_*, and what more expedient sequence of conditions gives values that are closest to *correct.* This paper also relates the hysteresis observed in temperature and angle sweeps to that observed in field sweeps. These relationships can be used to estimate how much *I_c_* hysteresis will be observed in different materials or different sequences of conditions using a limited data set.

Two commercially produced multifilamentary HTS tape specimens^1^ were studied: Ag-matrix (Bi,Pb)2Sr_2_Ca2Cu3O_10−_*_x_* (Bi-2223) and AgMg-matrix Bi_2_Sr_2_CaCu2O_8+*x*_ (Bi-2212). Out of all the HTS materials, these two are commercially produced in the largest quantities. Both specimens were considered state-of-the-art in 1999. Measurements were made as a function of magnetic field (0 T to 8 T), angle (−90° to 90°), and temperature (4 K to 80 K). For the Bi-2223 specimen, significant *I_c_* hysteresis was observed in field, angle, and temperature sweeps. Much less hysteresis was observed in measurement on the Bi-2212 specimen under all conditions. References [6], [7] reported results on Bi-2212 and Bi-2223 with field and angle sweeps at 4 K that are complementary to the results presented here. This paper adds hysteresis data at other temperatures and with temperature sweeps. The addition of the temperature dimension allows for a generalization that angle-sweep and temperature-sweep data are related to the hysteresis observed when the field is swept up and down at constant angle and temperature.

The source of this hysteresis and the difference between Bi-2223 and Bi-2212 is thought to be weaker intergrain coupling and the larger effective field at the grain boundaries in Bi-2223, which is (a) enhanced with increasing field by the diamagnetically excluded flux from the superconductive grains, and (b) lowered with decreasing field by the trapped flux inside the grains [1], [4]. The effect of hysteresis on *I_c_* may depend on many factors such as: materials processing, magnetic field strength, field angle, temperature, criteria (see below), and extent of field sweep. As these materials evolve, the magnitude of the hysteresis effects may change.

Clearly, *I_c_* is one of the most important parameters for characterizing a superconductor. In the transport current method for *I_c_* measurements, the voltage drop along the conductor is measured as a function of transport current through the conductor, which is referred to as the conductor’s voltage-current (*V*–I) characteristic or curve. *I_c_* is determined by applying a criterion to the measured *V*–I curve. All functional *I_c_* definitions are based on an *I_c_* criterion and the measurement of a finite voltage drop along the superconductor. The two most commonly used *I_c_* criteria are the electric-field strength (often referred to as just electric field) criterion *E_c_* and the resistivity criterion *ρ_c_.* Typical values of *E_c_* are 0.1 µV/cm or 1 µV/cm. Typical values of ***ρ****_c_* vary from 10^–8^ **Ω *·*** cm to 10^–12^ **Ω *·*** cm, depending on the current density and measurement sensitivity. Currently the 1 µV/cm criterion is most frequently used for HTS materials; however, the 0.1 µV/cm criterion has been specified for some magnet applications. The *V–I* curve of a superconductor can be modeled by the approximate empirical equation:

$$V\;=V_{0}\cdot {\left(I/I_{0}\right)}^{n},$$ (1)

where the *n*-value reflects the abruptness of the transition from the superconducting state to the normal state and *V*_0_ and *I*_0_ are constants. Typical *n*-values for Bi-based tapes are 5 to 50; typical Bi-2223 specimens have *n*-values near the high end of this range and typical Bi-2212 specimens have *n*-values near the low end of this range. The estimated expanded uncertainty (coverage factor *k* = 2, and thus a two-standard-deviation estimate) of the critical-current measurement is **±**2 %±0.2 A. The estimated expanded uncertainty of the angle measurement is **±**2**°**. Most of the *I_c_* measurements reported here were made using an electric-field strength criterion of 0.1 µV/cm. Some of the *I_c_* data at 1 µV/cm are provided on the Bi-2212 specimen because the *n*-values are low, which causes the critical currents at the two criteria to differ significantly. Often the measured *I_c_* is converted into a critical-current density *J_c_* by dividing the *I_c_* by some cross-sectional area of the conductor. This cross-sectional area may be one of the following: total conductor, only the superconductor, or the total conductor less any material added as stabilizer (e.g., copper, silver). *J_c_* determined using the cross-sectional area of the total conductor is often referred to as the engineering critical-current density, *J_e_.*

## 2. Procedure

### 2.1 Apparatus

**Fig. 3:**
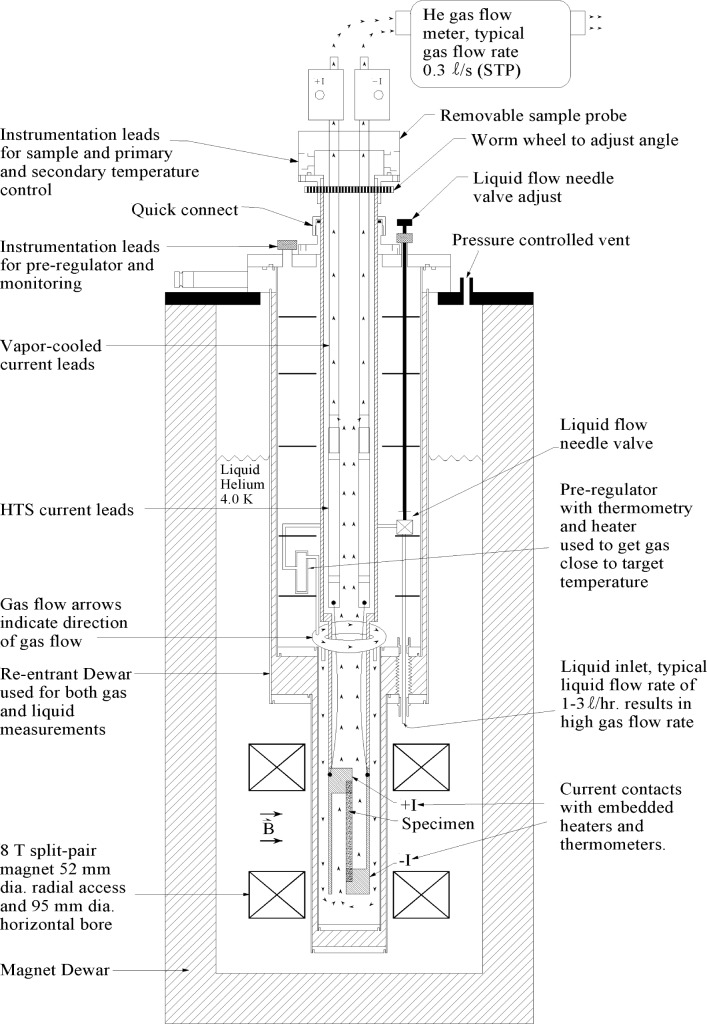
Cross-sectional schematic (not to scale) of the variable-temperature cryostat illustrating the basic design components.

The variable-temperature cryostat used in this study is a research device and a detailed description is beyond the scope of this paper. However, a few important details need to be mentioned. Fig. 3 is a cross-sectional schematic of the variable-temperature cryostat illustrating the basic design components. This is a helium gas-flow cryostat, which can also be filled with liquid helium. The cryostat is inserted into the 52 mm diameter radial-access (vertical) port of an 8 T split-pair magnet with a 95 mm diameter (horizontal) bore. The helium gas-flow rate can be adjusted with a typical rate of 0.3 L/s, at standard temperature and pressure, providing a temperature range of 4.2 K to 120 K. When operating with liquid helium, the cryostat can be sealed from the background magnet Dewar. The cryostat may be pressurized and the liquid heated to come into thermal equilibrium at temperatures as high as 5 K. Whether the cryostat is operated with gas or liquid, the background magnet operates near 4.02 K, which is the normal boiling point of liquid helium at the (reduced) atmospheric pressure of this test site (1650 m elevation above sea level). For the remainder of this paper, the terms “liquid” and “gas” are used to refer to the state of helium in which the specimen is immersed.

Since the accuracy of high-current *I_c_* measurements made when the specimen is in a gaseous environment is more problematic than measurements made in a liquid cryogen, a comparison of liquid and gas *I_c_* data was needed to determine the accuracy of variable-temperature measurements. A low-temperature superconductor (LTS) Cu/Nb-Ti specimen was selected for this comparison since it has a higher temperature dependence, more stable critical currents, and less *I*_c_ hysteresis than a HTS specimen would have. With the selection of a LTS specimen, the comparison between liquid and gas measurements needs to be made between 4 K and 5 K in helium. The measurements at these lower temperatures are the most difficult because the heat capacity of the specimen and the apparatus is very low, thus small amounts of heat can create large temperature changes. In addition, the higher critical currents at the lower temperatures are more challenging. Therefore, we have chosen to perform measurements on a LTS (Cu/Nb-Ti) specimen in helium as a definitive test of the accuracy of this HTS cryostat.

**Fig. 4:**
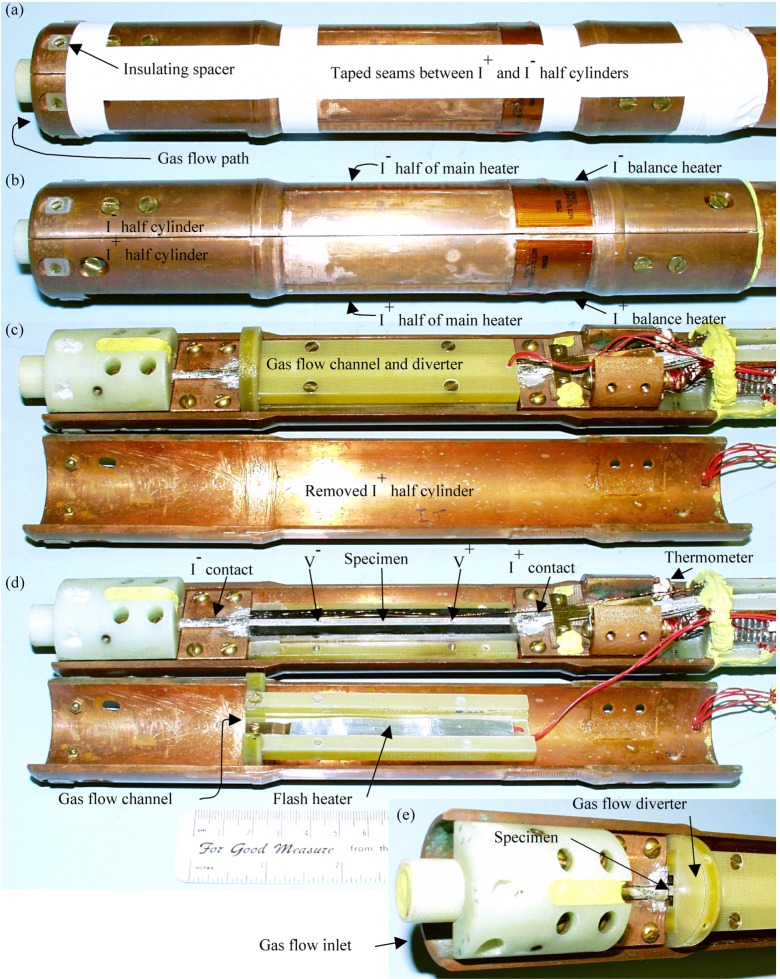
Images of specimen mounting area of variable-temperature cryostat: (a) full assembly, (b) without tape, (c) with I+ half cylinder removed, (d) with gas-flow channel and diverter removed (approximate scale given in (d)), (e) view of bottom gas inlet.

The salient features of the variable-temperature cryostat are: a primary temperature control of one specimen current contact with the heater split equally between the two specimen current contacts, a secondary control circuit that keeps the two specimen contacts at the same temperature using separate balance heaters on each current contact, a preregulator control circuit that converts the in-flowing helium liquid to gas and warms the gas to near the target specimen temperature, and gas flow that is routed over the current contacts and specimen. Figure 4 has images of the specimen mounting area of the variable-temperature cryostat in different states of assembly. The two specimen current contacts and the gas-flow path were designed to be as symmetric as possible to give them similar cooling conditions. The contacts were also designed to be as large as possible with extended surface areas for the gas to flow over. The thermal masses of each contact were also balanced. The secondary control uses one thermometer in each current contact and software that sends an analog signal to a dual heater-control circuit that delivers power to either of the two balance heaters. Typically, this power is about 0.1 W to 0.3 W. The preregulator software monitors the primary specimen heater power and adjusts the temperature set point of the preregulator heater to attempt to achieve a target power for the primary specimen heater. A typical target for the primary specimen heater power is 0.35 W. With this target and a helium gas-flow rate of about 0.3 L/s (monitored at standard temperature and pressure), the typical preregulator heater power is about 1 W for a specimen temperature range of 4 K to 10 K. This relatively high gas-flow rate is considered necessary to achieve a more uniform temperature along the length of the specimen between the current contacts. The helium gas-flow rate is lowered progressively for the higher temperatures to reduce the cooling power, and a typical value is about 0.1 L/s for temperatures of 50 K and higher. The preregulator heater power in creased with temperature and is typically more than 2 W for temperatures higher than 50 K. The purpose of the preregulator is to preregulate the gas temperature, which allows for more precise temperature control of the entire length of the specimen. Additional details are not included here since this apparatus is a research device incorporating many features not necessary for routine measurements.

A separate heater is used to rapidly warm the center part of the specimen, not the entire apparatus, so that the specimen can be efficiently reset to the virgin state. This procedure is referred to as “flash heating” and is used to greatly decrease the length of time necessary for cooling. This heater has a tape geometry (0.86 cm **×** 8.8 cm) and is located just above and co-planar to the tape specimen with a gap of about 2 mm. This gap is one of the He gas-flow channels. In order to monitor the specimen temperature, the *V–I* characteristic was measured during the flash heating. The heater was left on until the *V–I* characteristic is ohmic and the effective *I_c_* at 1 µV/cm is less than 0.04 A, which is less than 0.004 A at 0.1 µV/cm. With this procedure, the maximum specimen temperature may be slightly below *T_c_.* The flash heater power was about 14 W for the Bi-2223 *(T_c_* = 110 K) specimen and about 10 W for the Bi-2212 *(T_c_* = 85 K) specimen. A lower power level was used for the Bi-2212 specimen because of the lower *T_c_* and to reduce the thermal stress on the specimen. In each case, the specimen could be driven normal while most of the rest of the apparatus was still below 45 K or 50 K. Considering that the low-temperature heat capacity is proportional to *T^3^*, this meant that the cooling requirement was less than one-eighth of that necessary if the entire apparatus had to be warmed to near 100 K. Steady-state measurements of the normal-state *V–I* characteristic determined an effective *I_c_* of about 0.002 A at 0.1 µV/cm for both HTS specimens. With this procedure, the Bi-2223 specimen could be heated from 4 K to near its *T_c_* and cooled back to 4 K in less than 15 min. Each of the specimens was cycled to near or above its *T_c_* more than 60 times. Some of these cycles were a gradual warming overnight, but most were done with the flash heater. We compared the zero-field critical currents measured after flash heating to that measured after cooling the entire apparatus from a temperature above 120 K, and there was less than a 1 % difference for both HTS specimens. The “start” arrow on many of the plots presented here indicate the first measurement after the specimen was flash heated or cooled from a temperature higher than 120 K.

### 2.2 Thermometry

Temperature control for the measurements can be achieved using a resistive thermometer, which has a low magnetoresistance effect, or a capacitance thermometer, which has a very low magnetoresistance effect. However, for this study, the need for precise control and precise measurements precluded the use of a capacitance thermometer. Because a capacitance thermometer cannot be calibrated, it can be used only by setting the temperature at zero field using a calibrated thermometer, and then controlling the temperature with the capacitance thermometer as the field is swept. This method is not efficient for acquiring data at many temperatures and one field. The data presented here were taken using a resistive thermometer to achieve precise control, precise measurement, and efficiency. To maintain the most direct comparison of liquid and gas data, no magnetoresistance correction was applied to the temperature data. The estimated corrected temperature would have been within 10 mK for fields up to 5 T and 35 mK higher at 8 T. The variation of the measured temperature, with the angle of the applied field from −90° to 90° at 8 T, was less than 5 mK at 4 K.

### 2.3 Mandrel and Specimens

The specimen mandrel consisted of a straight stainless steel (AISI type 316) beam with copper current contacts on each end. The cross-section of the stainless steel beam was 0.635 cm × 1.27 cm. The active length (distance between the current contacts) of the specimens was about 10 cm. The voltage tap separation was 5 cm. All measurements were made with the specimens mounted in a straight-specimen geometry along the radial-access bore of the magnet.

The LTS specimen, which was used as a definitive test of the accuracy of this HTS cryostat, was a commercial multifilamentary Cu/Nb-Ti wire with a diameter of 0.76 mm and a Cu to Nb-Ti ratio of about 7 to 1. The LTS specimen and a copper ribbon were both soldered to the mandrel with non-superconducting solder. The copper ribbon was added to provide extra stability for the LTS specimen, and to provide a cooling surface, a total cross-sectional area, and a *J_e_* that were more similar to those of the tape HTS specimens. The copper ribbon was 0.1 mm by 5.1 mm. The Nb-Ti wire had an *I_c_* of 436 A at 1 T, 4 K, and 0.1 µV/cm, with an *n*-value of 41. Thus, the *J_e_* (using the total cross-sectional area of the conductor and the Cu ribbon) was 449 A/mm^2^ at 1 T. The measured normal-state resistance of the mounted specimen and mandrel was 5.5 µω at 10 K and 0 T; thus, the effective *I_c_* with the specimen in the normal state would be about 0.09 A. This represents a relatively low level of current sharing, which would not significantly bias the measurements.

The HTS specimens were mounted on the stainless steel portion of the mandrel with a glass-filled epoxy and were soldered to copper current contacts. A copper ribbon was not used in the mounting of the HTS specimens. The Bi-2223 specimen had a cross section of 0.24 mm × 3.9 mm and a *I_c_* of 251 A (*J*_e_ = 272 A/mm^2^) at 0 T, 4 K, and 0.1 µV/cm, with an *n*-value of 54. The Bi-2212 specimen had a cross section of 0.21 mm × 3.0 mm and a *I_c_* of 137 A (*J*_e_ = 220 A/mm^2^) at 0 T, 4 K, and 0.1 µV/cm. The low-field *n*-value for the Bi-2212 specimen was 5 to 6 for temperatures of 65 K and below. The Bi-2212 specimen had an *I_c_* of 225 A at 0 T, 4 K, and 1 µV/cm.

### 2.4 Data Acquisition

**Fig. 5:**
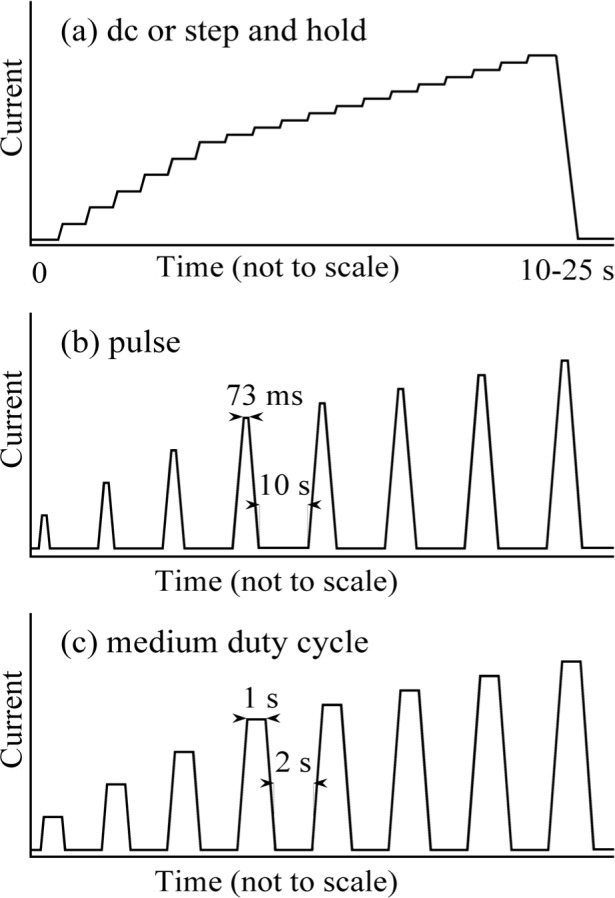
Illustration of the typical data acquisition patterns of current versus time: (a) dc or step and hold, (b) pulse, (c) medium-duty cycle.

General information about the procedure for determination of *I_c_* is discussed in Ref. [3]. The medium-duty-cycle method used for obtaining the voltage-current characteristics *(V–I* curve) was designed to reduce the temperature rise from lead, contact, and specimen heating. It involves delivering a series of discrete, approximately 1 s duration, current pulses to the conductor with a recovery time between pulses, and then increasing the amplitude of each successive pulse. Figure 5 is an illustration of the typical data acquisition patterns of current versus time. The current pulse has a trapezoidal shape with time; that is, it starts at zero, ramps up at a finite and relatively constant rate, holds, and then ramps back to zero. Voltage and current readings are acquired before, during, and after each pulse. Analysis of these readings was used to correct the thermoelectric voltages over a short time period. Most of the data presented in this paper were taken with this medium-duty-cycle method. This medium-duty-cycle method is a compromise between the pulse and dc methods. The pulse method minimizes heating and thermoelectric voltages but does not have good measurement sensitivity and there can be problems with specimen motion. In the pulse method, the current is pulsed, with typical durations less than 100 ms and very fast ramp rates. Large inductive voltages and short settling times can lead to a biased and varying signal. The dc method does have good measurement sensitivity with little specimen motion but does not minimize heating and thermoelectric voltages. The dc method is a step-and-hold method, similar to the medium-duty-cycle method but without the current returning to zero between the current levels, which results in a stair-step pattern of current versus time. Two variations on the dc method, slow dc and fast dc, were used. The slow dc had a longer settling time after each current ramp and the meter readings were made over a longer time interval. Measurements made using these different techniques were compared to determine whether agreement could be achieved.

## 3. Results

### 3.1 Measurement Verification

**Fig. 6:**
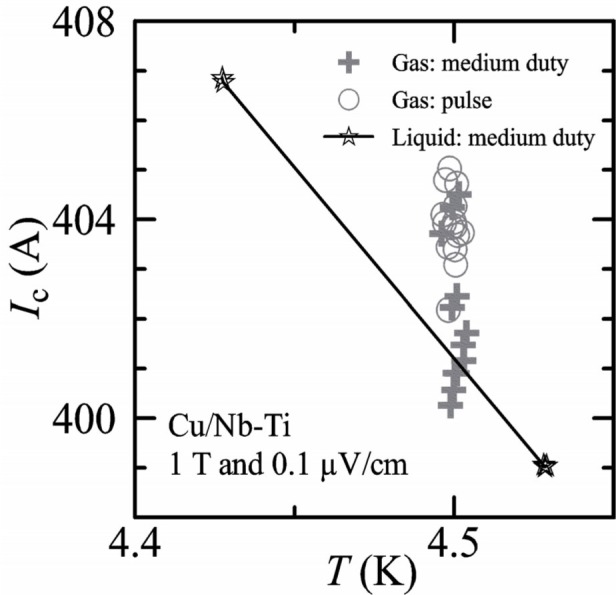
versus temperature at 1 T of a Cu/Nb-Ti specimen. Measurements were made with different acquisition methods and in both liquid and gaseous helium environments. The legend indicates the method and whether the measurements were made in liquid or gas.

*I_c_* measurements were made in liquid and gas at various temperatures on a Cu/Nb-Ti specimen to verify measurements in gas to currents over 400 A. Figure 6 shows *I_c_* data at 0.1 µV/cm over a narrow temperature range in a magnetic field of 1 T. The data points represented by the star symbol were taken in liquid with the medium-duty-cycle method. There are three determinations of *I_c_* at each of two temperatures (although the individual symbols fall nearly on top of each other), which are on each side of the gas data near 4.5 K. A linear fit line connects the liquid data and is a good approximation for the temperature dependence of *I_c_* in this region. The data points represented by the plus symbol (medium-duty) and the circle symbol (pulse) were taken with the specimen in flowing helium gas. The uppermost gas data point is less than 50 mK from the line determined by the liquid data, which suggests that the temperature error is less than 50 mK. The observed variation in the repeat determinations of the gas data was significantly higher than for the liquid data. This is likely due to small variations in the difference between the thermometer and the specimen temperatures. The average *I*_c_ value for the pulse data in gas is above the liquid determined line and this suggests that the specimen is slightly cooler than the thermometer. This apparent supercooling of the specimen relative to the thermometer was observed during the development of the hardware and operating conditions of the apparatus. Some cooling power at the specimen is necessary to handle varying heat loads and to balance the temperature of the two current contacts; however, too much cooling power causes the average specimen temperature to be lower than the current contacts and thermometer. This was the motivation for the relatively low-target specimen heater power. The primary improvement in the liquid/gas comparison during the evolution of the apparatus was achieved by lowering the target power of the specimen heater, which lowered the amount of supercooling. The average *I*_c_ value for the medium-duty data in gas is above, but closer to the liquid determined line, and this is consistent with a little more specimen heating than in the pulse data in gas. The results obtained from the medium-duty and pulse methods were compared at other temperatures and magnetic fields. These results were used to check results in regions where a liquid-to-gas comparison was not possible. In all cases, similar temperature uncertainties were observed.

**Fig. 7:**
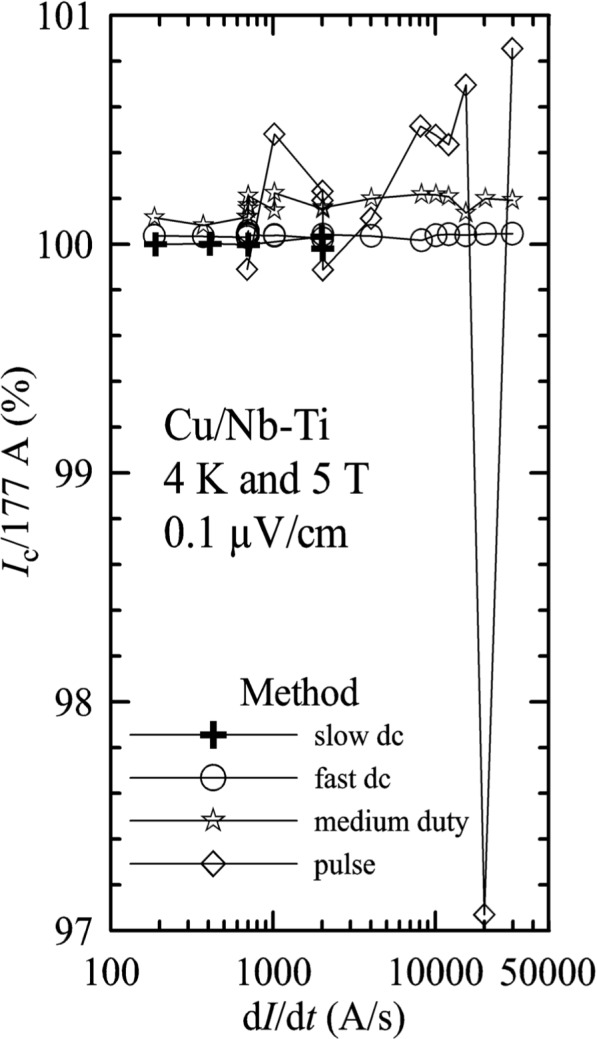
Normalized *I*_c_ versus current-ramp rate for the Cu/Nb-Ti specimen at 4 K and 5 T with measurements made using different acquisition methods.

A verification of acquisition methods and current ramp rates was conducted on the Cu/Nb-Ti wire. Figure 7 is a semi-logarithmic plot of normalized *I_c_* at 0.1 µV/cm versus current ramp rate, d*I*/d*t*, at 4 K (in liquid He) and 5 T for various data acquisition methods. All of the *I_c_* determinations were normalized by a single value of approximately 177 A. A connecting line was drawn through the determinations for each method to aid the reader. The variability of the pulse method determinations was larger than the other methods. Systematic differences of less than 0.2 % were observed in the non-pulse methods. These differences arise from differences in the locations of the voltage-current setpoints and from which points are fitted to determine *I_c_.* Some additional voltage noise was observed in the medium-duty and pulse methods for ramp rates above 10 kA/s. This was thought to be due to a slight vibration of specimen in the magnetic field that resulted from the quick application of the Lorentz force on the specimen during the current ramp. This explanation was supported by the fact that less voltage noise was observed in lower Lorentz force measurements to over 400 A at 1 T with ramp rates up to 30 kA/s. Most of the measurements reported in this paper were made with current ramp rates between 700 A/s and 4 kA/s. Additional verifications of acquisition methods and current ramp rates were conducted on both HTS specimens, and no significant differences were observed.

### 3.2 Hysteresis With Magnetic Field Sweeps

#### 3.2.1 Bi-2223

Figure 2a is a semi-logarithmic plot of *I_c_* versus magnetic field for Bi-2223 tape at 4 K and a field angle of 90°. The measurements in zero applied magnetic field (only in self-field) were assigned an arbitrary value of 0.01 T to allow them to be plotted on the logarithmic scale. The measurements at 0.02 T are fairly consistent with this field assignment. A start arrow points to the first value at zero field. There are four curve segments shown: first up, first down, second up, and second down. These curve segments form the two branches of the hysteresis loop that are determined by the sweep direction of the magnetic field. *I_c_* was measured in a number of fixed magnetic fields and the sweep direction refers to whether the field had been increased or decreased to reach that setpoint. The points at the extremes of the sweep are plotted with multiple symbols to close the loops. These data show that *I_c_* at a given magnetic field (2 T) can be about 40 % higher when it is measured with monotonically decreasing fields (from a higher field) compared to monotonically increasing fields. Thus, magnet overshoot, drift, or specimen motion could lead to significant errors. The largest difference between the sweep direction occurs at about 2 T to 3 T. The two curves taken with increasing magnetic fields do not converge until the field is 1 or 2 T. For the second loop, the highest field was 3 T. The two curves taken with decreasing magnetic fields are nearly the same for all fields. The second loop indicates that the 3 T field needs to be reduced to about 1 T to complete the switch from one branch to the other. The quick change from one branch to the other means that overshooting a field could mix the results from the two branches. As with most hysteretic phenomenon, the value at the highest field point is repeated on each cycle. The initial/virgin branch is not repeated unless the specimen is warmed to near or above its *T*_c_ and then cooled in zero field. Also, the best chance of repeating a value on the initial sweep up, without warming the specimen, is accomplished by ramping the field to zero, then increasing to the desired setpoint.

Hysteresis is also observed on this same Bi-2223 specimen with the magnetic-field angle at 0°, as shown in Fig. 2d at 4 K. The specimen was flash heated before the initial point indicated by the start arrow. These curves are similar to the curves at 90° in Fig. 2a. The measurements in zero magnetic field were again assigned a value of 0.01 T to allow them to be plotted on the logarithmic scale. The measurements at 0.02 T are fairly consistent with this field assignment. The *I_c_* measured at zero magnetic field after a magnetic field sweep is lower than the initial *I_c_* measured at zero field by about 9 %. The largest difference between the sweep direction occurs at about 0.2 T and is about 74 %. The two curves taken with increasing magnetic fields converge at a field of about 0.2 T, which is much lower than the equivalent point for the 90° data. The two curves taken with decreasing magnetic fields are nearly the same for all fields. The second loop indicates that the 3 T field needs to be reduced to 2 T to complete the switch from one branch to the other, which is faster than observed at 90°.

Similar hysteresis curves are shown in the remaining plots in Fig. 2 at both angles and temperatures of 20 K and 35 K. Again, the specimen was flash heated between each setting of temperature and angle. The size of the hysteresis decreases with increasing temperature. For the higher fields and temperatures, there is little or no hysteresis.

**Fig. 8:**
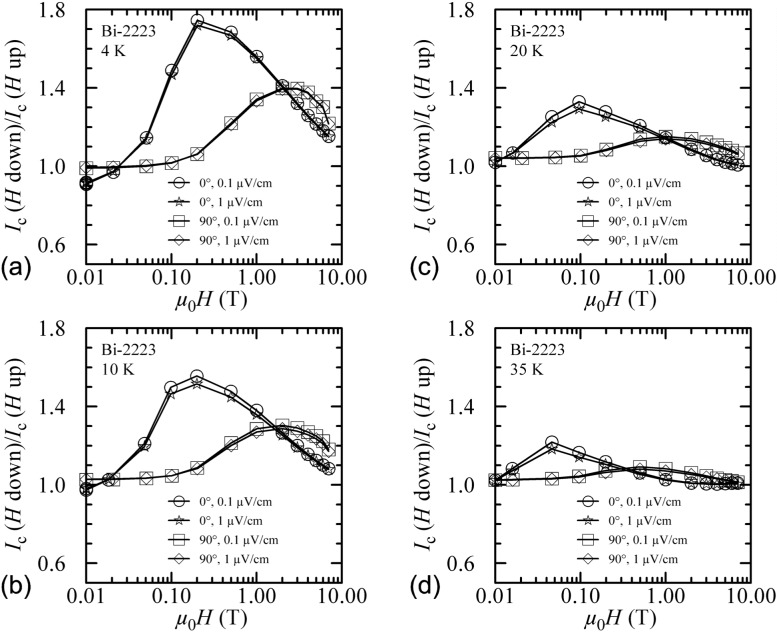
*I*_c_(*H* down)/*I*_c_(*H* up) versus magnetic field for the Bi-2223 specimen for various angles, criteria, and temperatures: (a) 4 K, (b) 10 K, (c) 20 K, (d) 35 K.

**Fig. 9:**
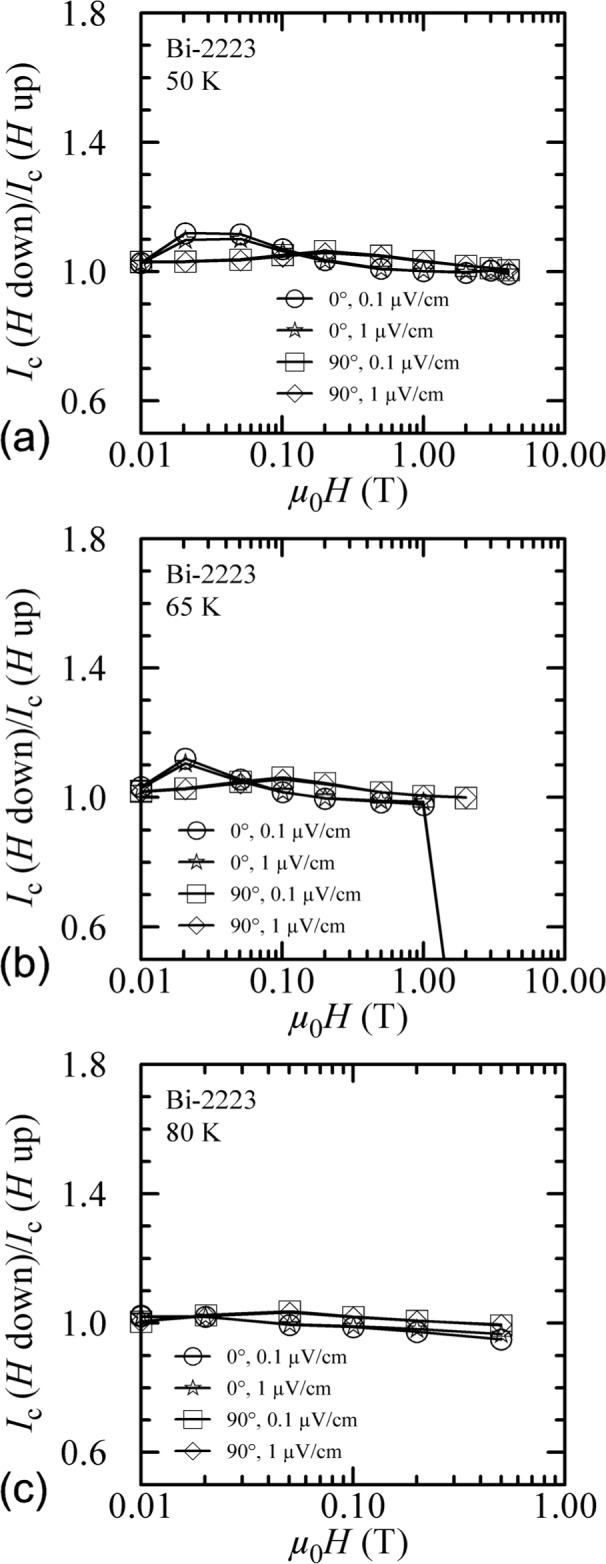
*I*_c_(*H* down)/*I*_c_(*H* up) versus magnetic field for the Bi-2223 specimen for various angles, criteria, and temperatures: (a) 50 K, (b) 65 K, (c) 80 K.

Another way to show the field-sweep hysteresis is to plot the ratio of *I_c_* measured with decreasing field to that measured with increasing field, *I_c_(H* down)/*I*_c_(*H* up), versus magnetic field. Such plots are shown in Figs. 8 and 9 at temperatures of 4 K, 10 K, 20 K, 35 K, 50 K, 65 K, and 80 K. In each plot, curves are shown for 0**°** and 90° and for 0.1 µV/cm and 1.0 µV/cm. The curves differ very little at different criteria. The anomalous ratios at the higher fields and temperatures (see Fig. 9b, 0°) are due to slight differences in the small *I_c_* values. The size and field dependence of the hysteresis is illustrated in these curves.

**Fig. 10:**
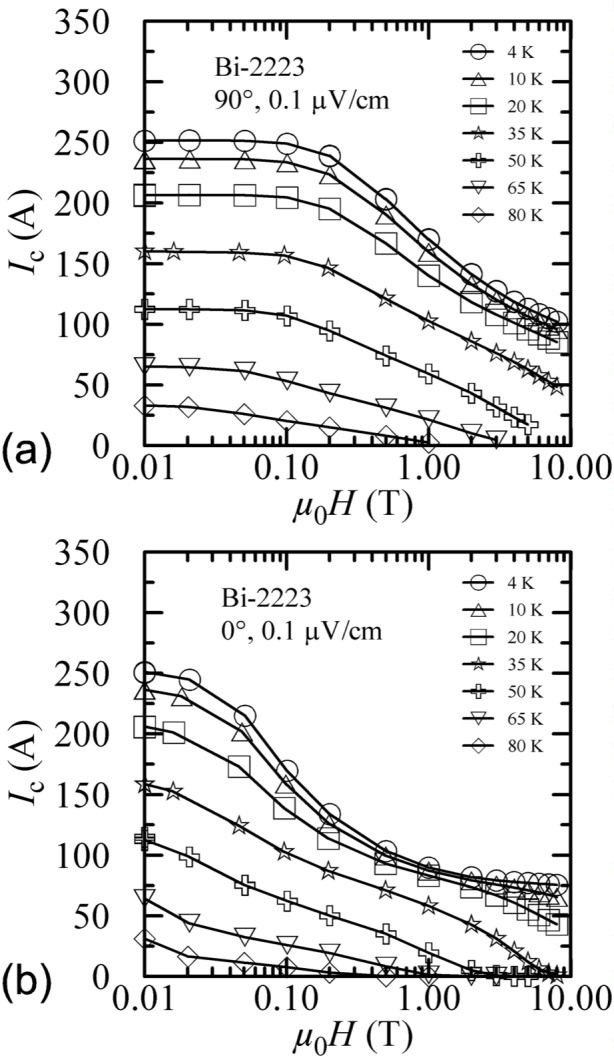
*I*_c_ at 0.1 µV/cm versus magnetic field (initial/virgin curves) for the Bi-2223 specimen for various temperatures and angles: (a) 90°, (b) 0°.

Temperature cross sections of the Bi-2223 specimen’s critical surface are shown in Fig. 10 at 90° and 0°. The curves shown were taken with increasing field. The specimen was flash heated before each curve. For each angle, the curves at different temperatures are similar. The curves at different angles differ. The *x*-axis is logarithmic to better illustrate the dependence over a wide range of magnetic fields. If the *x*-axis were linear, these cross sections of the critical surface would appear quite different.

#### 3.2.2 Bi-2212

**Fig. 11:**
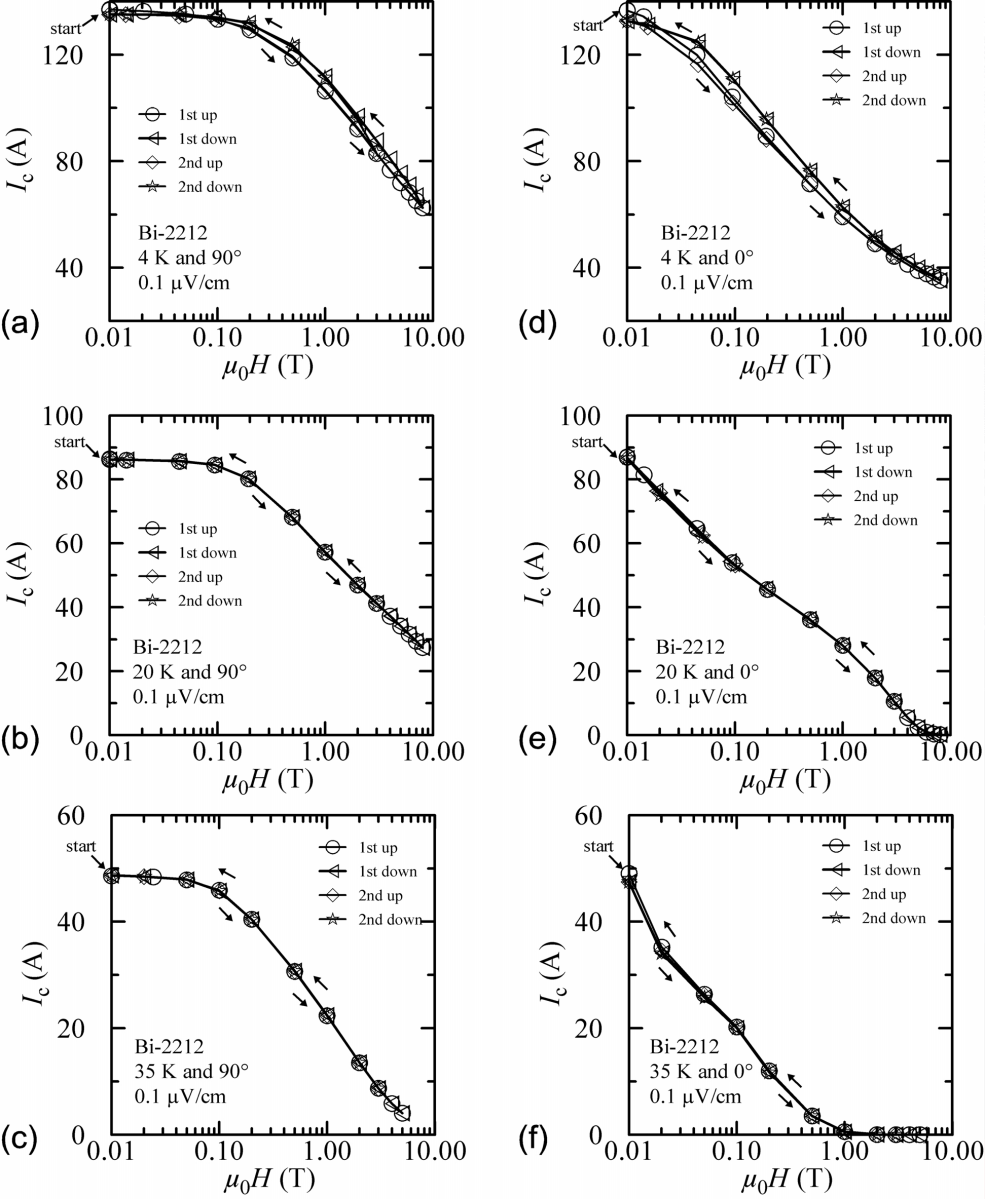
*I*_c_ at 0.1 µV/cm versus magnetic field for the Bi-2212 specimen for various field-sweep directions, temperatures, and angles: (a) 4 K and 90°, (b) 20 K and 90°, (c) 35 K and 90°, (d) 4 K and 0°, (e) 20 K and 0°, (f) 35 K and 0°.

Field-sweep hysteresis on the Bi-2212 specimen is shown in Fig. 11a at 4 K and 90°. The largest difference in measured *I*c occurs at about 3 T and is about 6 %. This is much less than the 40 % hysteresis observed in the Bi-2223 specimen at 4 K and 90°. For the Bi-2212 specimen, the largest difference between the first up and the second up is about 1 %. The difference between zero-field values is about 1.4 %.

Field-sweep hysteresis at 4 K and 0**°** is shown in Fig. 11d. The largest difference in measured *I_c_* occurs between 0.2 T and 0.5 T and is about 7 %. This is much less than the 74 % hysteresis observed in the Bi-2223 specimen at 4 K and 0°. For the Bi-2212 specimen, the largest difference between the first up and the second up is about 3 %. The difference between zero-field values is about 3 %.

**Fig. 12:**
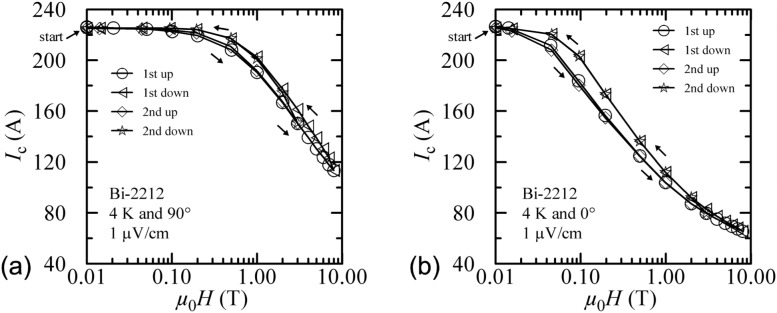
*I*_c_ at 1 µV/cm versus magnetic field for the Bi-2212 specimen at 4 K for various field-sweep directions and angles: (a) 4 K and 90°, (b) 4 K and 0°.

Figure 12 shows the hysteresis at 4 K and 1.0 µV/cm and both angles. Since the *n*-value of the Bi-2212 specimen is quite low, the *I_c_* values at this higher criterion are much larger than at 0.1 µV/cm. However, the observed hysteresis is very similar.

The rest of the plots in Fig. 11 show the field-sweep hysteresis at both angles and temperatures of 20 K and 35 K. Again, the specimen was flash heated between each setting of temperature and angle. There is very little hysteresis at 20 K and higher temperatures.

**Fig. 13:**
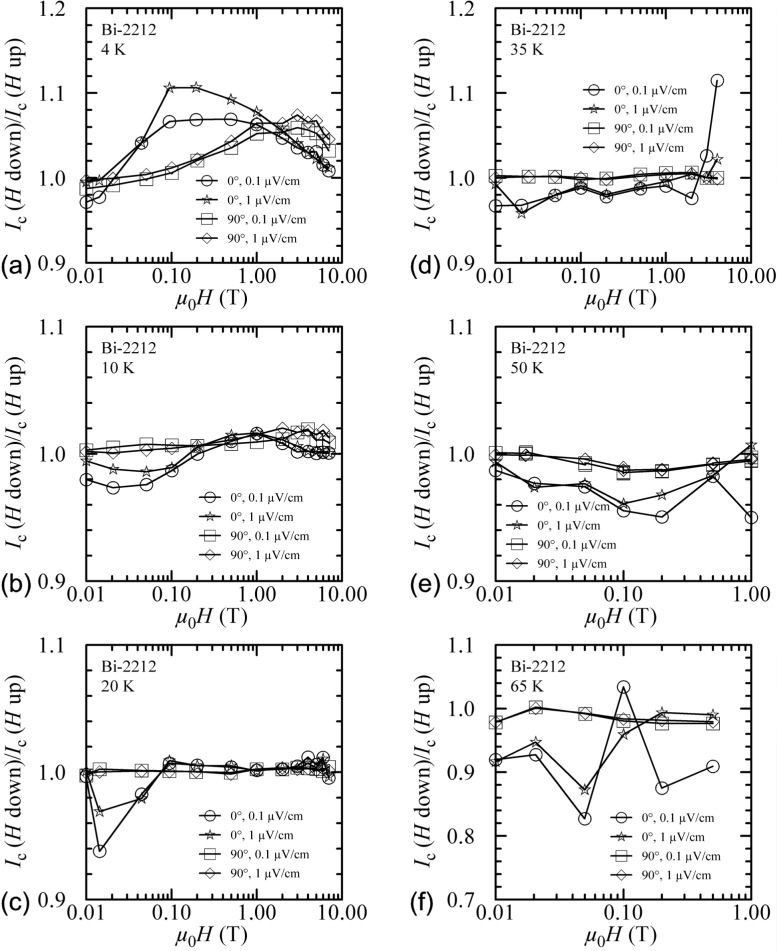
*I*_c_(*H* down)/*I*_c_(*H* up) versus magnetic field for the Bi-2212 specimen for various angles, criteria, and temperatures: (a) 4 K, (b) 10 K, (c) 20 K, (d) 35 K, (e) 50 K, (f) 65 K.

The plots of *I_c_(H* down)/*I*_c_(*H* up) versus magnetic field for the Bi-2212 specimen are shown in Fig. 13 at temperatures of 4 K, 10 K, 20 K, 35 K, 50 K, and 65 K. In each plot, curves are shown for 0**°** and 90° and for 0.1 µV/cm and 1.0 µV/cm. At 4 K and 0**°** there are some differences between the curves at different criteria, but otherwise there is little dependence on criteria. The anomalous ratios at the higher fields and temperatures (see Fig. 13f) are due to slight differences in the small *I_c_* values. The ratios at 1.0 µV/cm show less variability than those at 0.1 µV/cm.

**Fig. 14:**
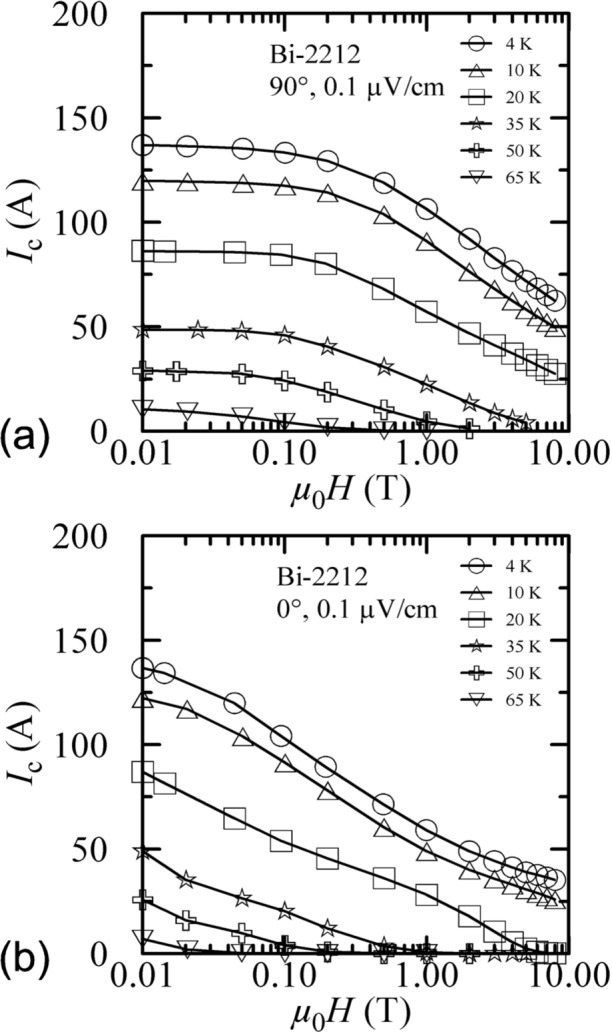
*I*_c_ at 0.1 µV/cm versus magnetic field (initial/virgin curves) for the Bi-2212 specimen for various temperatures and angles: (a) 90°, (b) 0°.

Temperature cross sections of the Bi-2212 specimen’s critical surface are shown in Fig. 14 at 90° and 0°. The curves shown were taken with increasing field. The specimen was flash heated before each curve. For each angle, the curves at different temperatures are similar. The curves at different angles differ.

### 3.3 Hysteresis With Angle Sweeps

#### 3.3.1 Bi-2223

Again, what are the *correct* values and procedures if hysteresis is observed in the measured *I*_c_ at different field angles? The *correct* method for measuring *I*_c_ should be dictated by the sequence of conditions experienced in an application. Furthermore, the *correct* method for measuring *I*_c_ at different field angles is analogous to that of the previous section dealing with field sweeps. Can an application expose a given segment of the magnet winding to selected field angles to improve the local conductor performance? Under the assumption that this cannot be achieved for most applications, the correct measurements are those obtained on the virgin or initial field sweep with the specimen held at a fixed angle for the entire curve. These will be referred to as fixed-angle field-sweep data. Acquiring the angle dependence by angle sweeps at a given field is expedient and common; thus we need to compare *I_c_* measurements acquired by these two techniques.

**Fig. 15:**
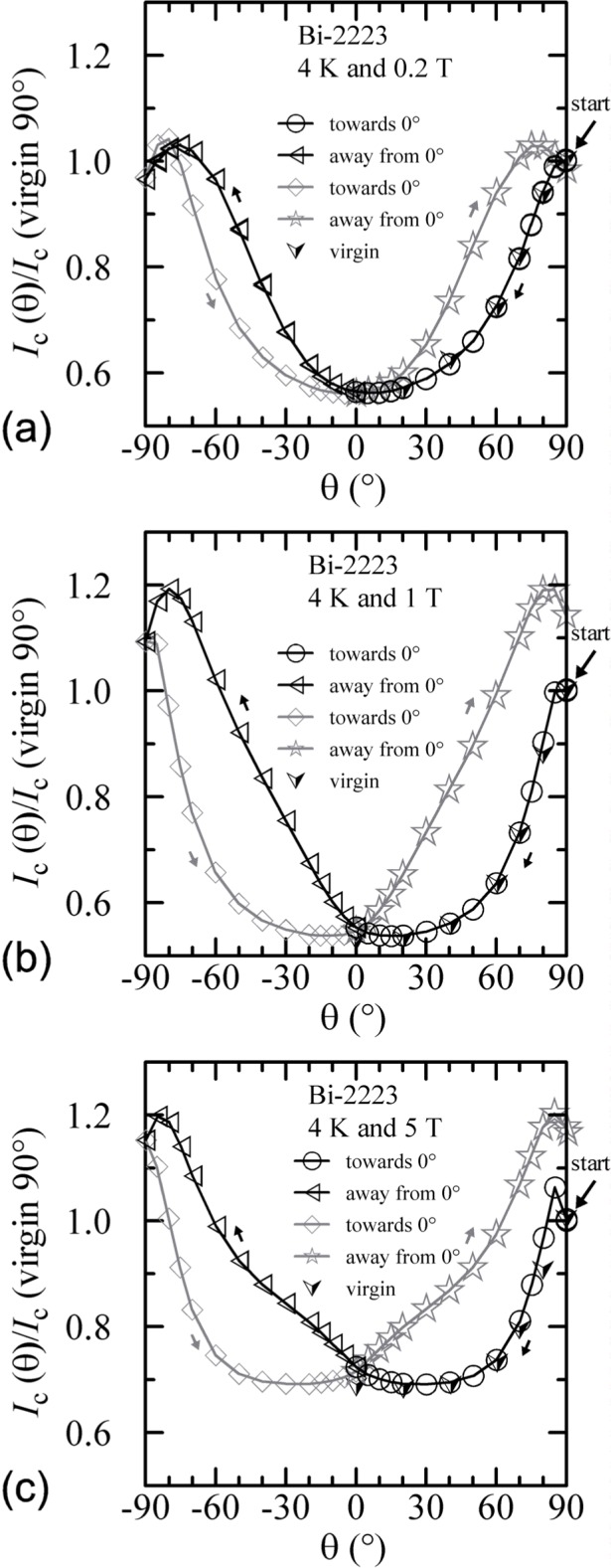
*I*_c_/*I*_c_(virgin 90°) at 0.1 µV/cm versus angle for the Bi-2223 specimen at 4 K for various sweep directions and magnetic fields: (a) 0.2 T, (b) 1 T, (c) 5 T.

The hysteresis observed on the Bi-2223 specimen with an angle sweep at 4 K and 5 T is shown in Fig. 15c. The starting point is the one indicated at 90° and was obtained with a fixed-angle field sweep, thus it is a virgin point. The *y*-axis is the *I_c_* normalized by the value at this starting point. Other fixed-angle field-sweep points are also shown with the virgin ([image]) symbol. The starting point is the only point along the angle-sweep curve that cannot be repeated without decreasing the field and flash heating. After the field angle has been swept and returned to 90°, the measured *I_c_* was about 17 % higher. Other than the first virgin point, the angle sweeps result in fairly reproducible hysteresis loops. For the angle-sweep data, the two branches of the hysteresis loops are determined by whether the angle is being swept towards 0**°** or away from 0°. The measured *I_c_* can be 32 % higher at a given magnetic-field angle depending on the angle-sweep direction. The minimum *I_c_* as a function of angle with sweep towards 0**°** occurs at about 30° and this value is nearly the same as the value obtained by a fixed-angle magnetic-field sweep at 0°. In other words, the *correct I_c_* at 0**°** happens to be the same as the minimum value obtained by an angle sweep. Thus, the apparent cusp near 0**°** is just an artifact of *I_c_* hysteresis. The angle-sweep *I_c_* at 0**°** is about 5 % higher than the fixed-angle field-sweep *I_c_.* The lower branches (sweep towards 0°) of the angle-sweep curve yield results that are closer to the *correct* fixed-angle data than the upper branches. A more general rule would be that the angle-sweep results are more *correct* when swept from higher *I_c_* to lower *I_c_.* Notice that this is the same rule as that followed in the field-sweep hysteresis.

**Fig. 16:**
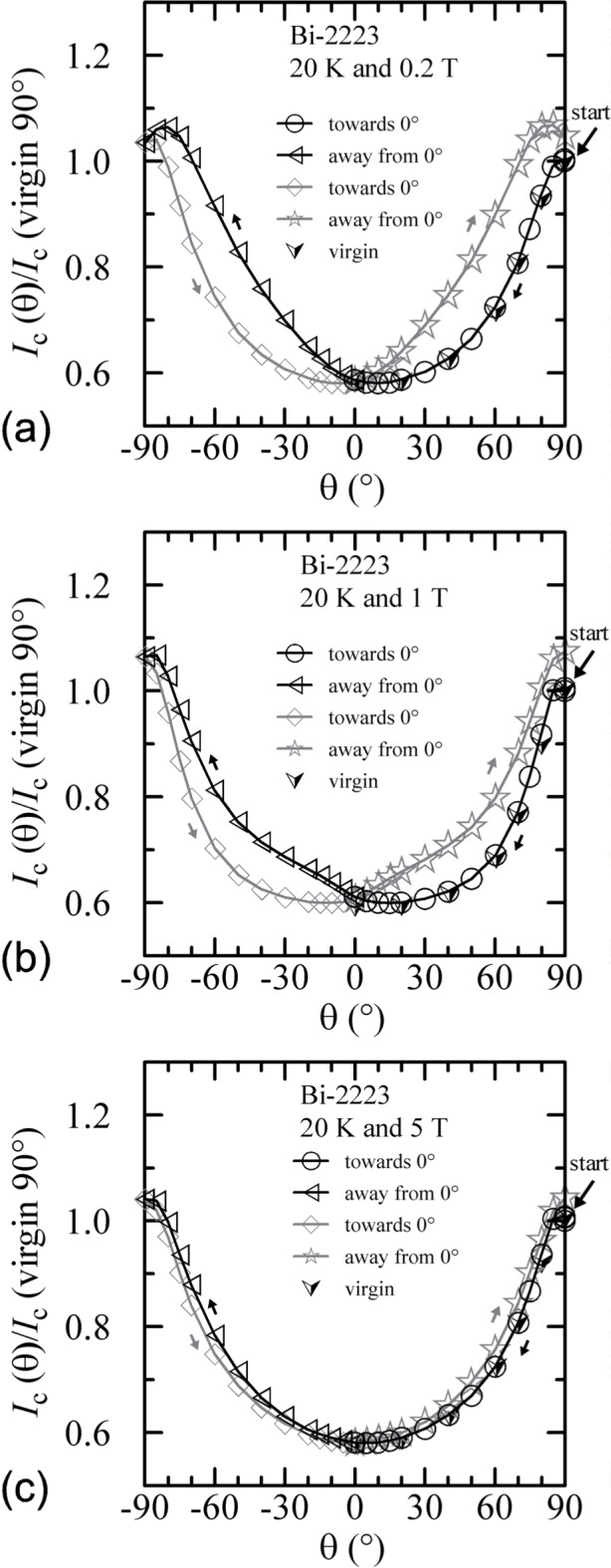
*I*_c_/*I*_c_(virgin 90°) at 0.1 µV/cm versus angle for the Bi-2223 specimen at 20 K for various sweep directions and magnetic fields: (a) 0.2 T, (b) 1 T, (c) 5 T.

**Fig. 17:**
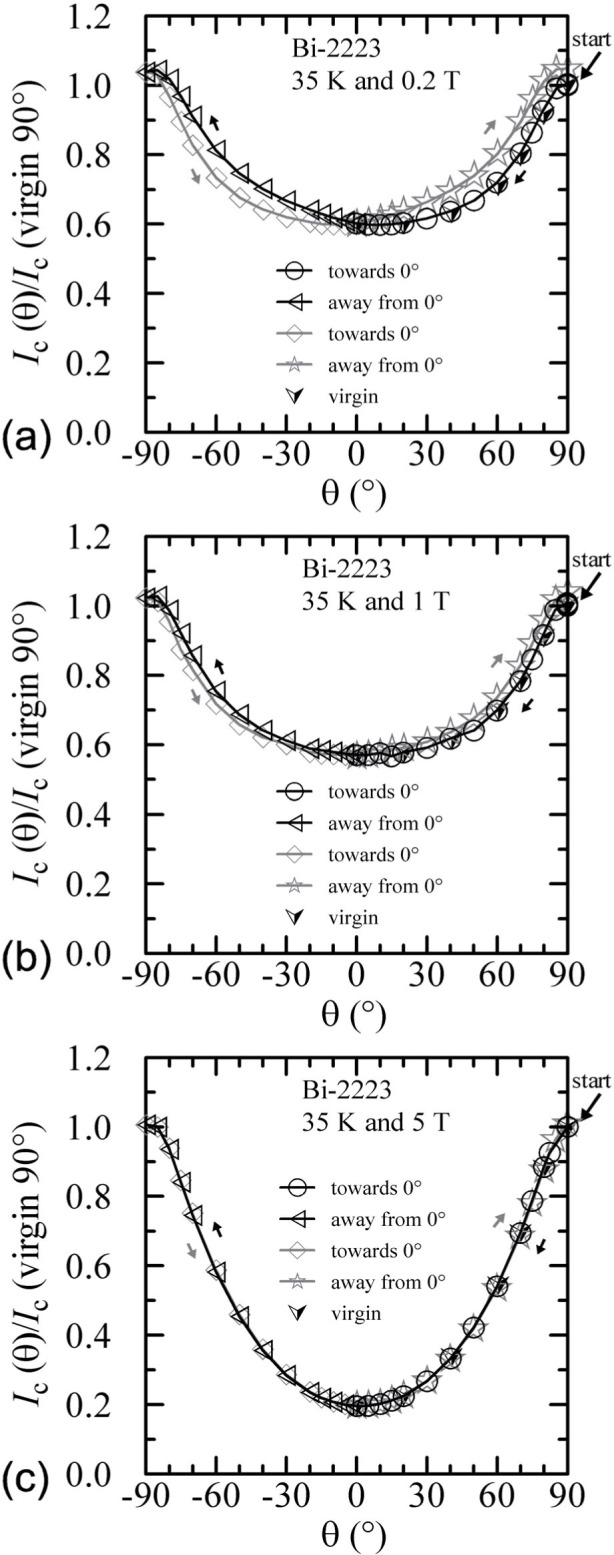
*I*_c_/*I*_c_(virgin 90°) at 0.1 µV/cm versus angle for the Bi-2223 specimen at 35 K for various sweep directions and magnetic fields: (a) 0.2 T, (b) 1 T, (c) 5 T.

Similar angle-sweep hysteresis was observed at 0.2 T and 1 T at 4 K, and systematically less hysteresis was observed at higher temperatures and all fields. Figures 15a, 15b, 16, and 17 show these angle-sweep and virgin data. The separation of the branches with sweep direction is significant for all measured fields at 4 K and for 0.2 T and 1 T at 20 K. There is very little hysteresis at 5 T and 20 K, and at 1 T and 5 T at 35 K. In all cases, the lower branches (sweep towards 0°) of the angle-sweep curve yield results that are closer to the virgin data than the upper branches. Also, the extra features (local maximum near 0**°** and extra bump when approaching 90°) decrease as the amount of hysteresis decreases. The largest difference between the virgin and swept data occurs at 90°.

**Fig. 18:**
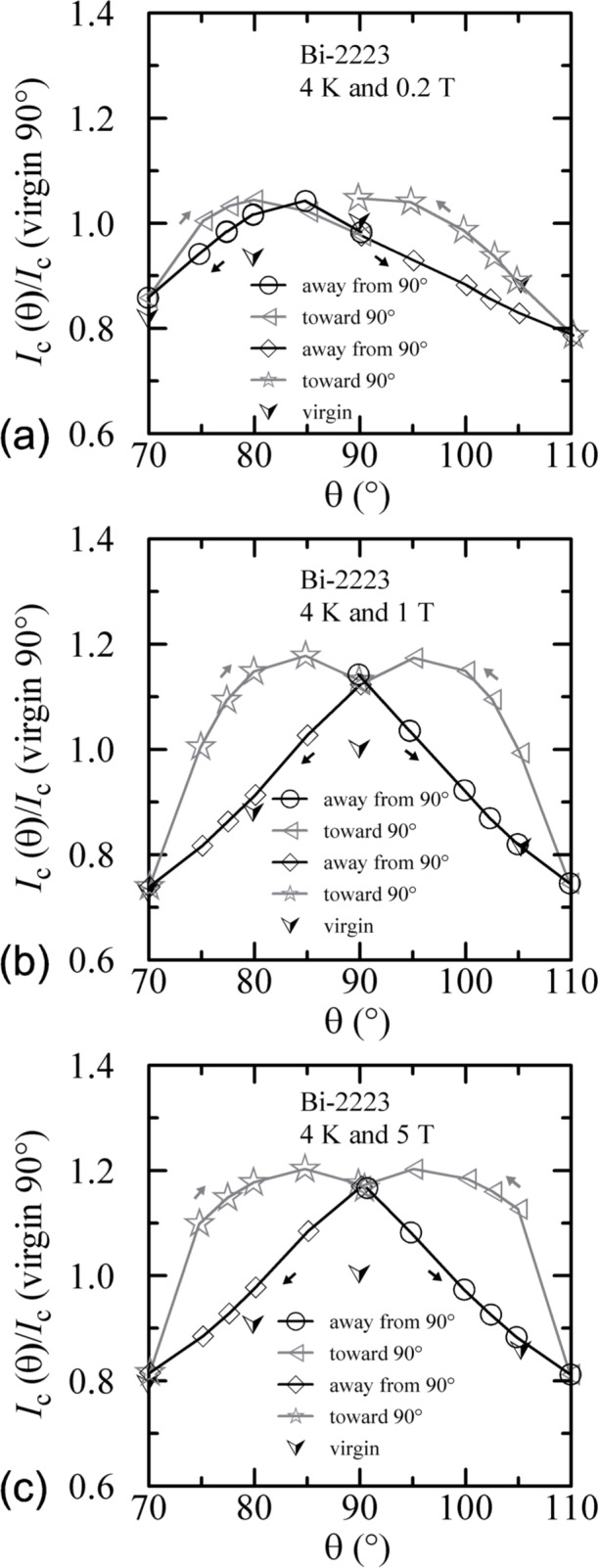
*I*_c_/*I*_c_(virgin 90°) at 0.1 µV/cm versus angle around 90° for the Bi-2223 specimen at 4 K for various sweep directions and magnetic fields: (a) 0.2 T, (b) 1 T, (c) 5 T.

**Fig. 19:**
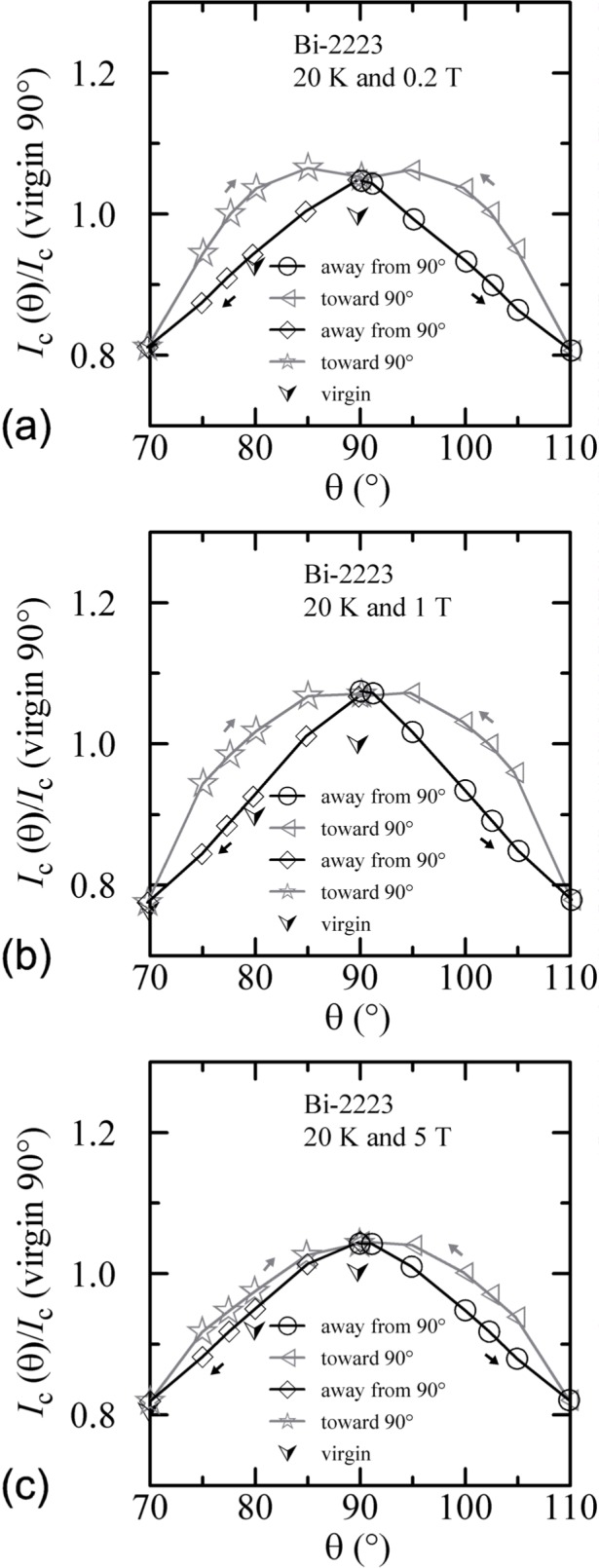
*I*_c_/*I*_c_(virgin 90°) at 0.1 µV/cm versus angle around 90 for the Bi-2223 specimen at 20 K for various sweep directions and magnetic fields: (a) 0.2 T, (b) 1 T, (c) 5 T.

**Fig. 20:**
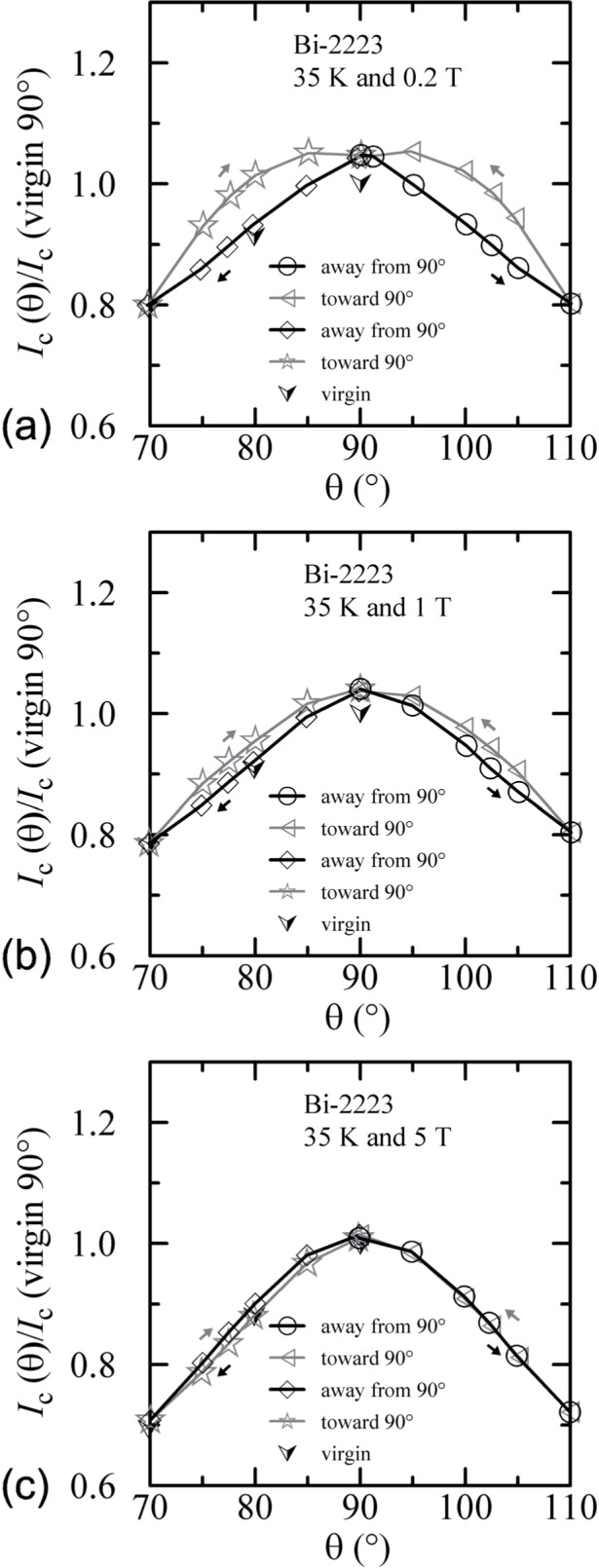
*I*_c_/*I*_c_(virgin 90°) at 0.1 µV/cm versus angle around 90° for the Bi-2223 specimen at 35 K for various sweep directions and magnetic fields: (a) 0.2 T, (b) 1 T, (c) 5 T.

An additional minor angle sweep around 90° was made after each of the previous sets of data. These minor sweeps are shown on Figs. 18–20. The branch sequence is given in the legend of each plot and the sequences are all the same except for Fig. 18a (4 K and 0.2 T). For most of the plots, the first branch was taken going from 90° to 110°, thus continuing in the same direction as the previous data, which was from −90° to 90°. The change in direction that occurred for the data in Fig. 18a may have shifted the first branch; however, some of the other branches seem slightly anomalous, which suggest that something may have gone wrong with these measurements. Typically, it was observed that a 5**°** change in angle was sufficient to switch from one branch to the other. The quick change from one branch to the other means that smooth rotation in one direction is needed to avoid mixing the results from both branches. Again the general rule is that the values from the lower branches are closer to the virgin values. The shape of the angle-sweep curves are not *correct* around 90°, the dependence on angle of the lower branches is enhanced compared to the virgin values. The peaks when approaching 90° from each side are nearly symmetric. As the amount of the hysteresis decreases, all branches approach the virgin values. Using the symmetry of virgin values for the 4 K plots and the sweep branches for all of these plots, the center of symmetry seems to be between 91° and 92°. This could be due to a slight error in the initial alignment of the cryostat.

**Fig. 21:**
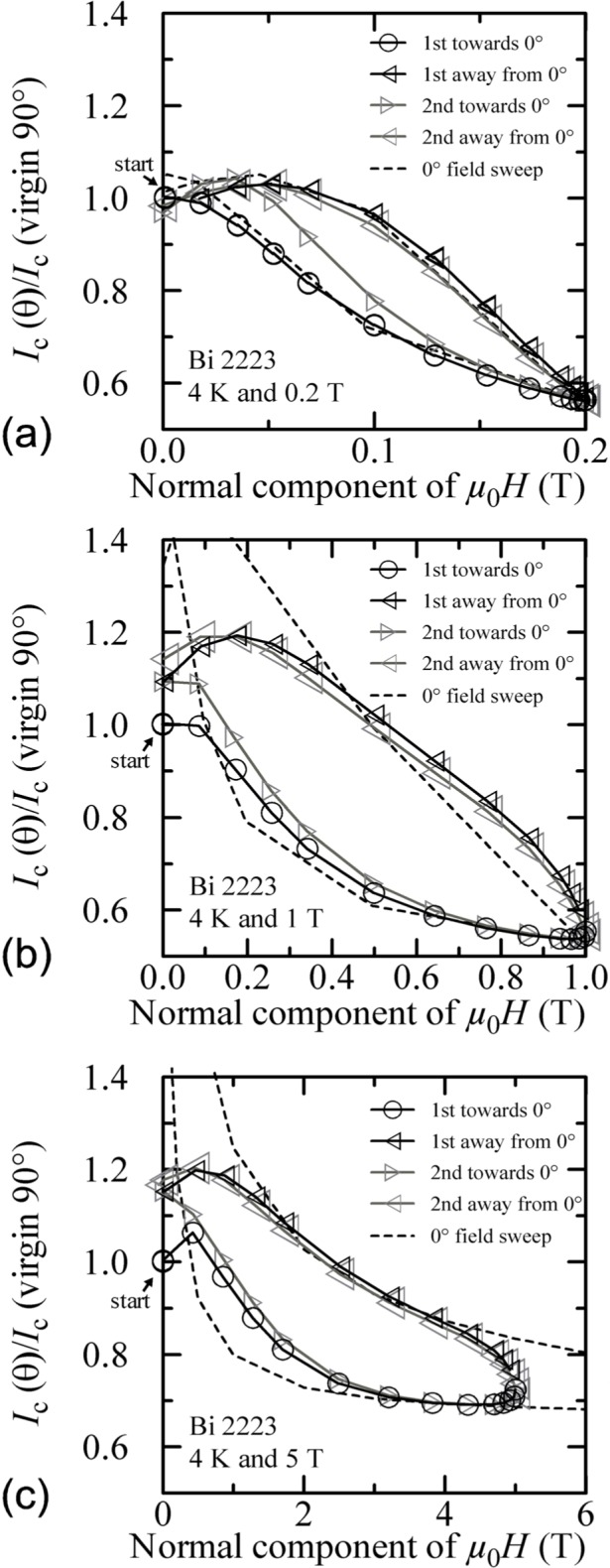
*I*_c_/*I*_c_(virgin 90°) at 0.1 µV/cm versus the normal component of the magnetic field for the Bi-2223 specimen at 4 K for the various angle sweeps and fields shown in Fig. 15: (a) 0.2 T, (b) 1 T, (c) 5 T. The dash curves show the *I*_c_ versus magnetic field curves at 0° taken from Fig. 2.

Reference [6] showed a correlation between the amount and nature of the hysteresis observed in magnetic field sweeps and that observed in angle sweeps at a given field. Following Ref. [6], another way to plot these angle-sweep data in Fig. 15 is shown in Fig. 21. The x-axis of Fig. 21 is the normal component of the magnetic field (*µ*_0_*H*cos *θ).* This folds the +90° and −90° data together and a single hysteresis loop is formed. The dashed hysteresis loop in Fig. 21c is the major loop of a field sweep from 0 T to 8 T at 0**°** using the same normalization value as used for the angle-sweep data. The dashed field-sweep hysteresis loops in Fig. 21a and 21b were minor loops from 0 T to 0.2 T and 0 T to 1 T, respectively. As observed in Ref. [6], when plotted in this way, most of the angle-sweep loop measured at 5 T (Fig. 21c) is closely approximated by the field-sweep loop measured at 0°, and there is a correlation between the amount of hysteresis observed in the angle-sweep loop and that observed in the field-sweep loop. The approximation departs from the 5 T angle-swept data at the lower fields. The approximation for the 0.2 T angle-swept data is much closer at all fields. This correlation suggests that the component of the field along 0**°** dominates the measured *I_c_.* This forms a good approximation except at the lower fields.

**Fig. 22:**
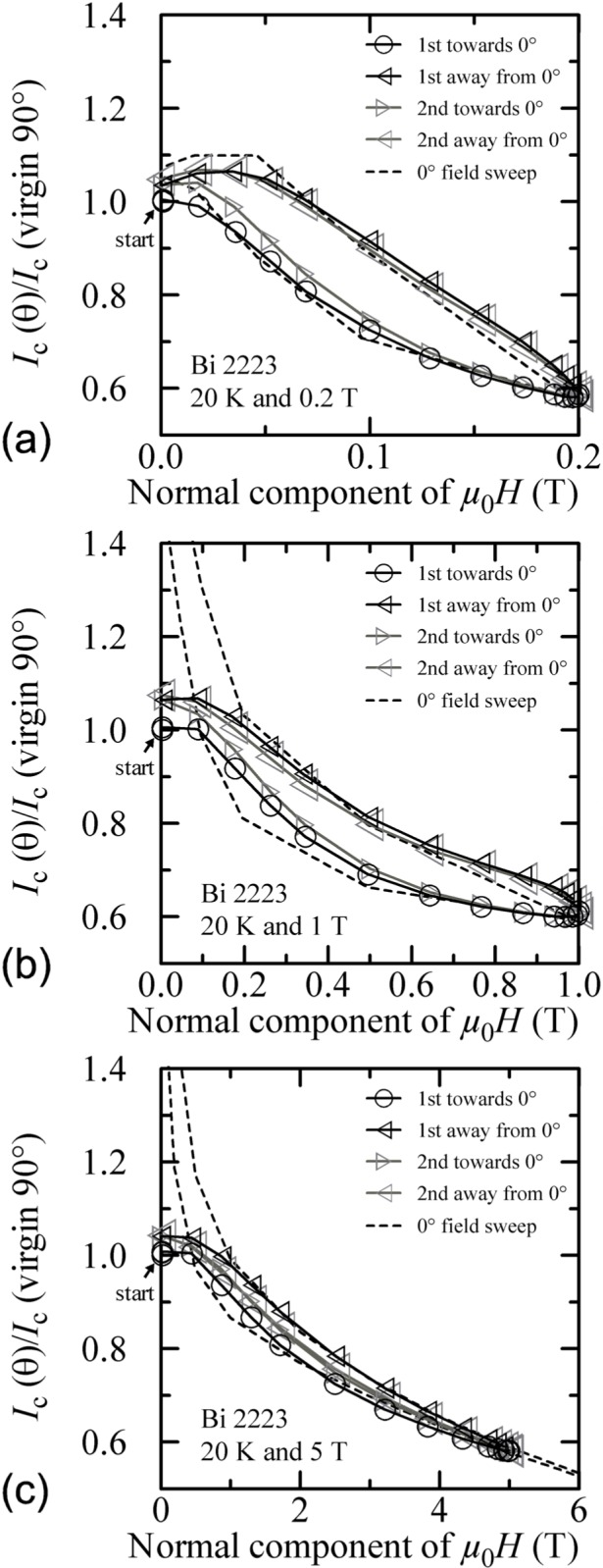
*I*_c_/*I*_c_(virgin 90°) at 0.1 µV/cm versus the normal component of the magnetic field for the Bi-2223 specimen at 20 K for the various angle sweeps and fields shown in Fig. 16: (a) 0.2 T, (b) 1 T, (c) 5 T. The dash curves show the *I*_c_ versus magnetic field curves at 0° taken from Fig. 2.

**Fig. 23:**
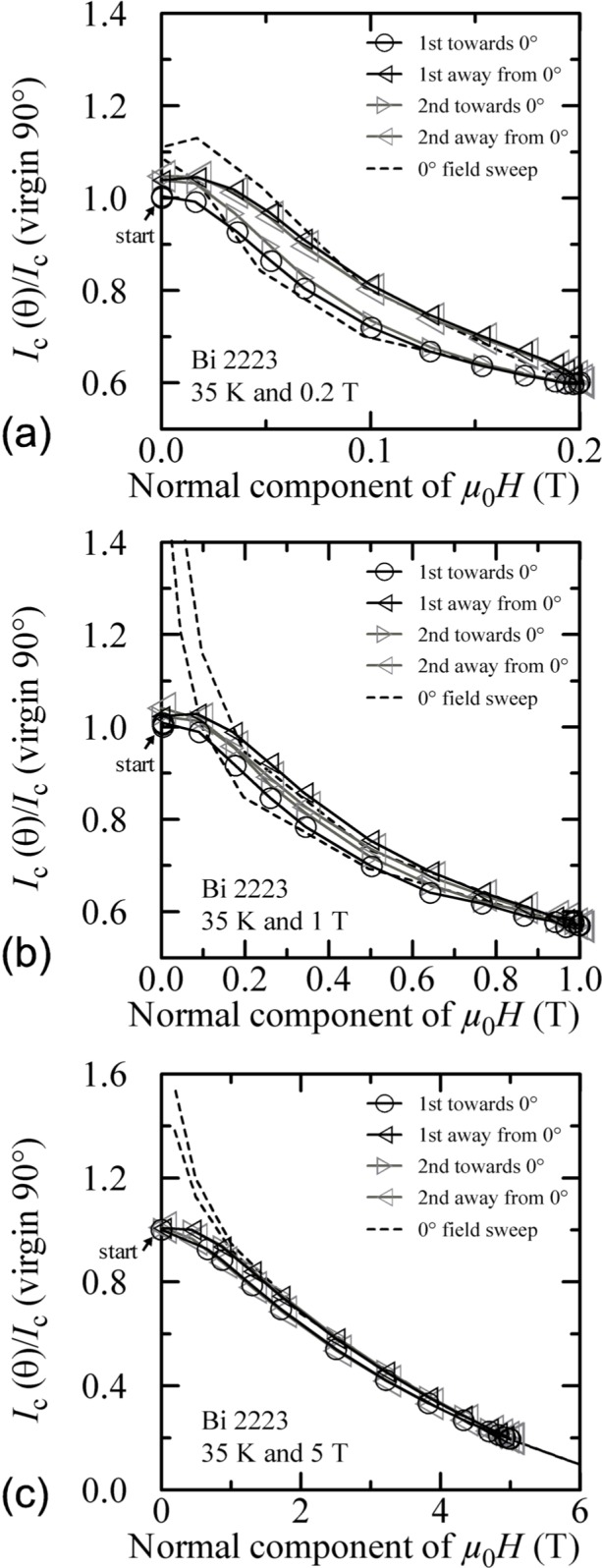
*I*_c_/*I*_c_(virgin 90°) at 0.1 µV/cm versus the normal component of the magnetic field for the Bi-2223 specimen at 35 K for the various angle sweeps and fields shown in Fig. 17: (a) 0.2 T, (b) 1 T, (c) 5 T. The dash curves show the *I*_c_ versus magnetic field curves at 0° taken from Fig. 2.

Additional plots of normalized *I_c_* versus the normal component of the magnetic field for other temperatures and fields are shown in Figs. 22 and 23. In each case, an appropriate 0**°** field-sweep data set is plotted as a dashed loop and is a good approximation to the hysteresis observed in the angle-sweep curves at a given field. These plots illustrate the strong correlation between the amount and nature of the hysteresis observed in field-sweep and angle-sweep data. The decrease in the observed hysteresis with increasing temperature and field is nearly the same for both types of data.

#### 3.3.2 Bi-2212

**Fig. 24:**
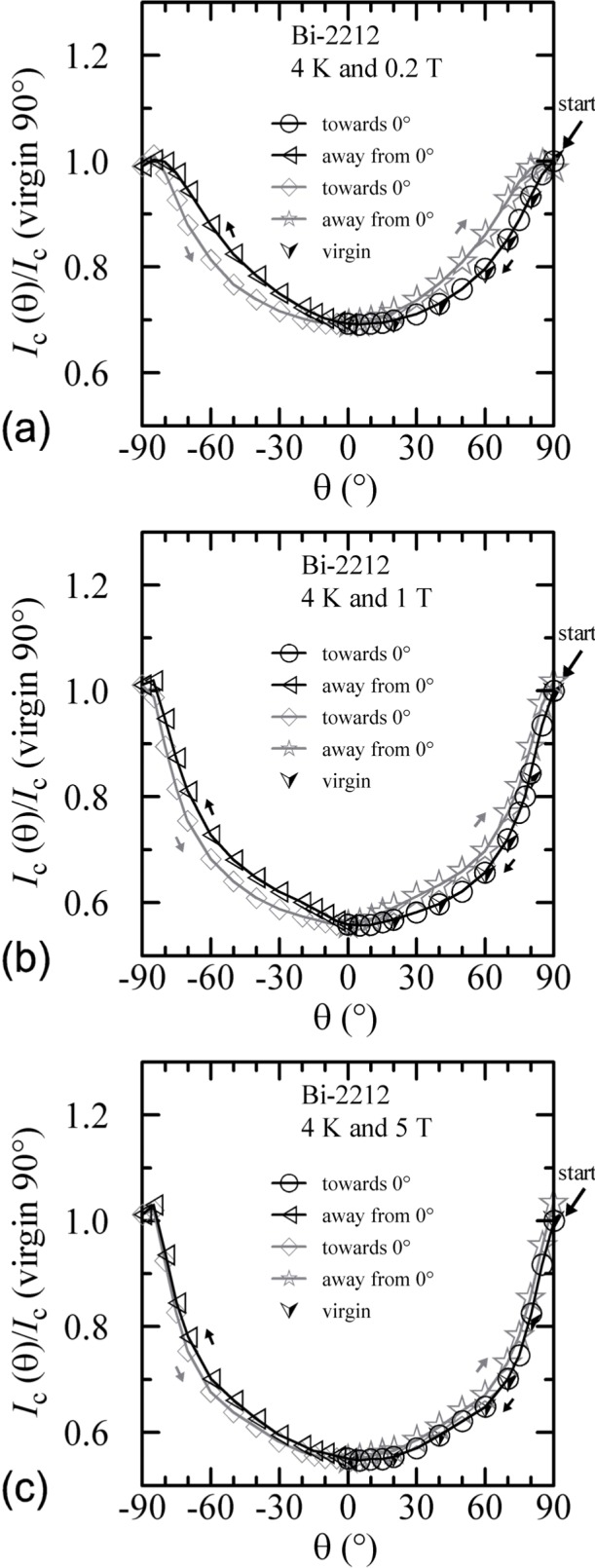
*I*_c_/*I*_c_(virgin 90°) at 0.1 µV/cm versus angle for the Bi-2212 specimen at 4 K for various sweep directions and magnetic fields: (a) 0.2 T, (b) 1 T, (c) 5 T.

**Fig. 25:**
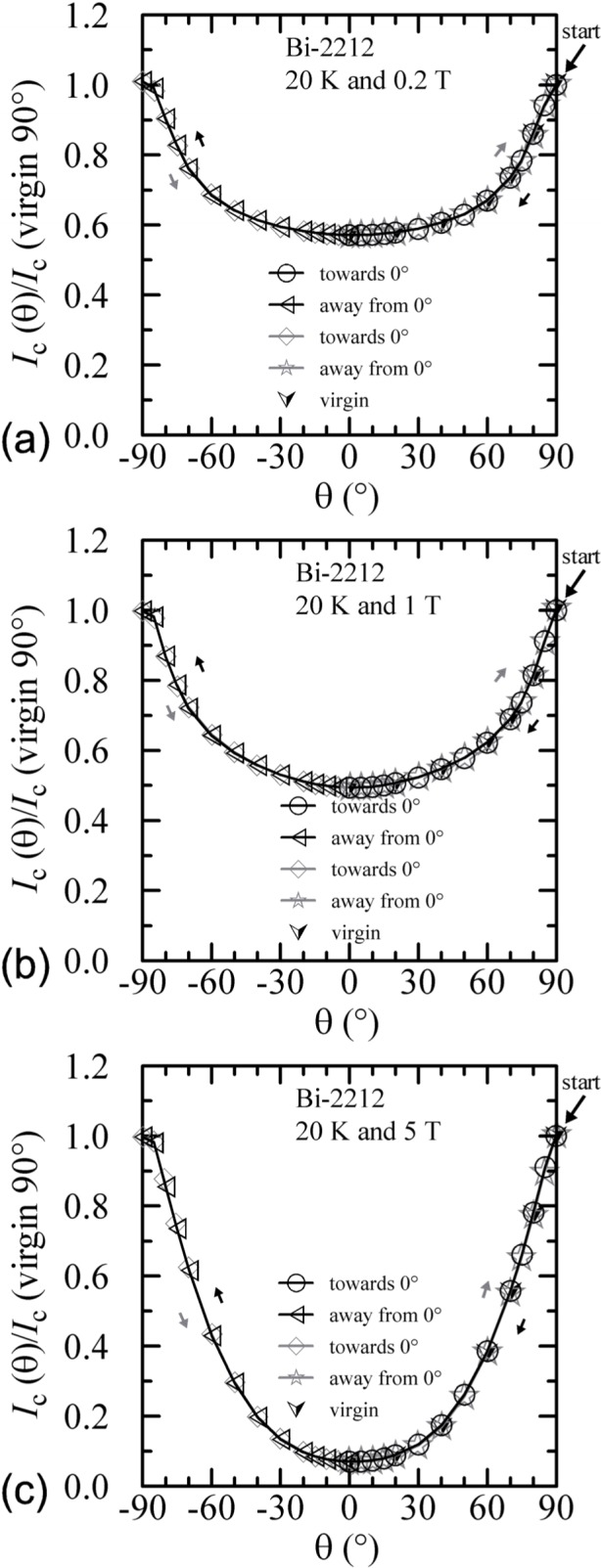
*I*_c_/*I*_c_(virgin 90°) at 0.1 µV/cm versus angle for the Bi-2212 specimen at 20 K for various sweep directions and magnetic fields: (a) 0.2 T, (b) 1 T, (c) 5 T.

**Fig. 26:**
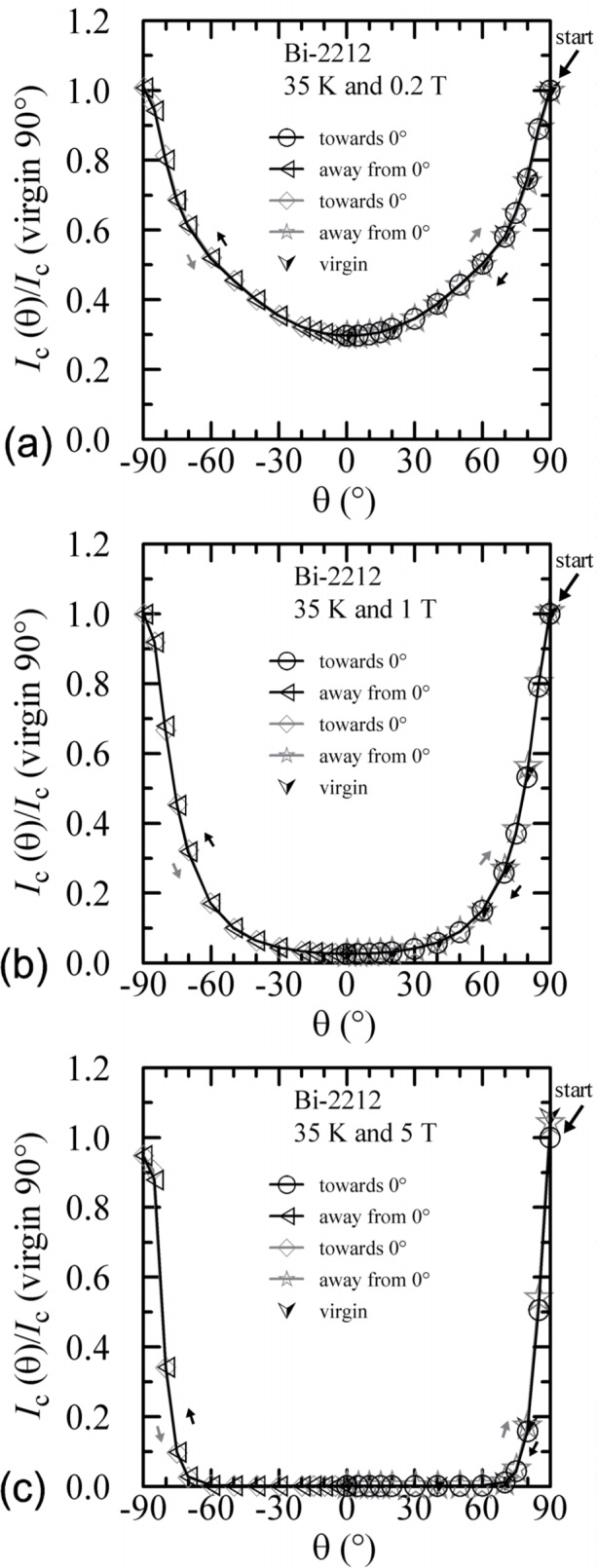
*I*_c_/*I*_c_(virgin 90°) at 0.1 µV/cm versus angle for the Bi-2212 specimen at 35 K for various sweep directions and magnetic fields: (a) 0.2 T, (b) 1 T, (c) 5 T.

The angle-sweep hysteresis observed on the Bi-2212 specimen was much less than that observed on the Bi-2223 specimen. Figures 24–26 show angle-sweep and virgin data on the Bi-2212 specimen. There is noticeable hysteresis only in the low-field data at 4 K. The higher-field and higher-temperature data have very little hysteresis. In all cases where there is hysteresis, the lower branches (sweep towards 0°) of the angle-sweep curve yield results that are closer to the virgin data than are the upper branches. The angular dependence at 5 T and 35 K was strong, with an *I_c_* of 3.8 A at 90°, and the specimen was normal *(I_c_* from 6 mA to 7 mA at 0.1 **µ**V/cm) for angles from −50° to 50°.

**Fig. 27:**
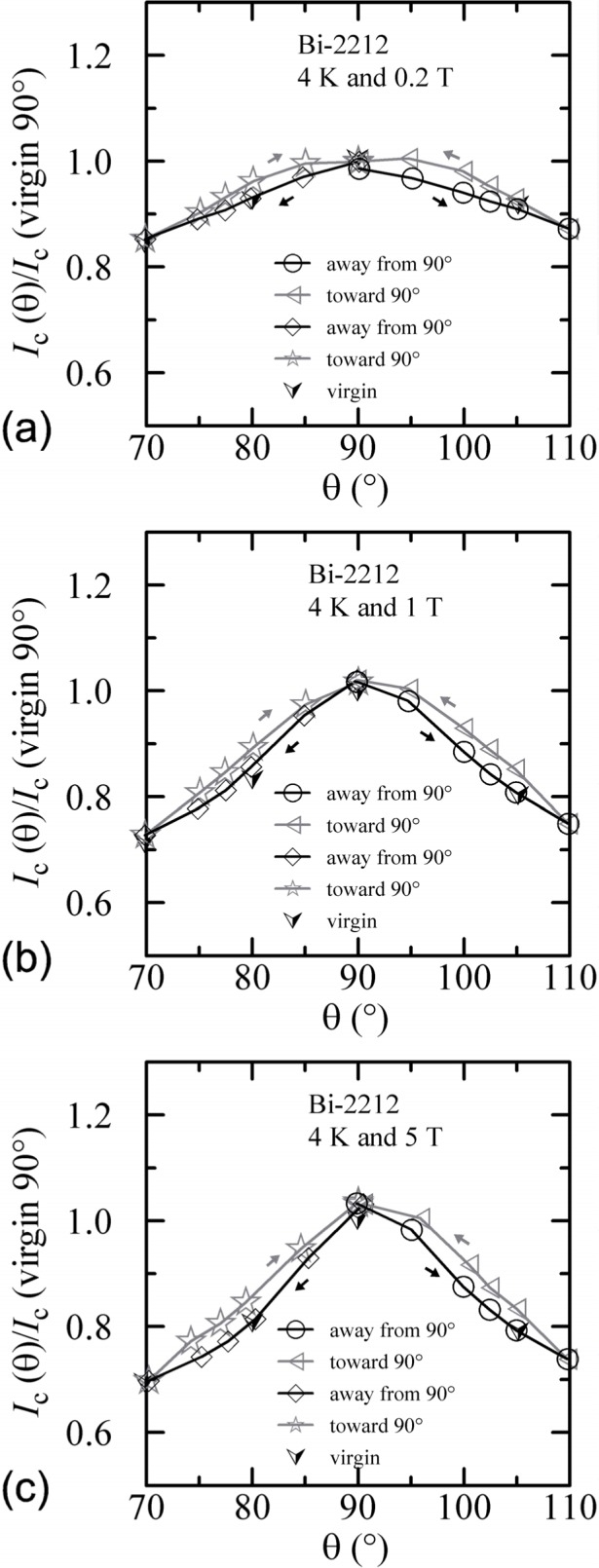
*I*_c_/*I*_c_(virgin 90°) at 0.1 µV/cm versus angle around 90° for the Bi-2212 specimen at 4 K for various sweep directions and magnetic fields: (a) 0.2 T, (b) 1 T, (c) 5 T.

**Fig. 28:**
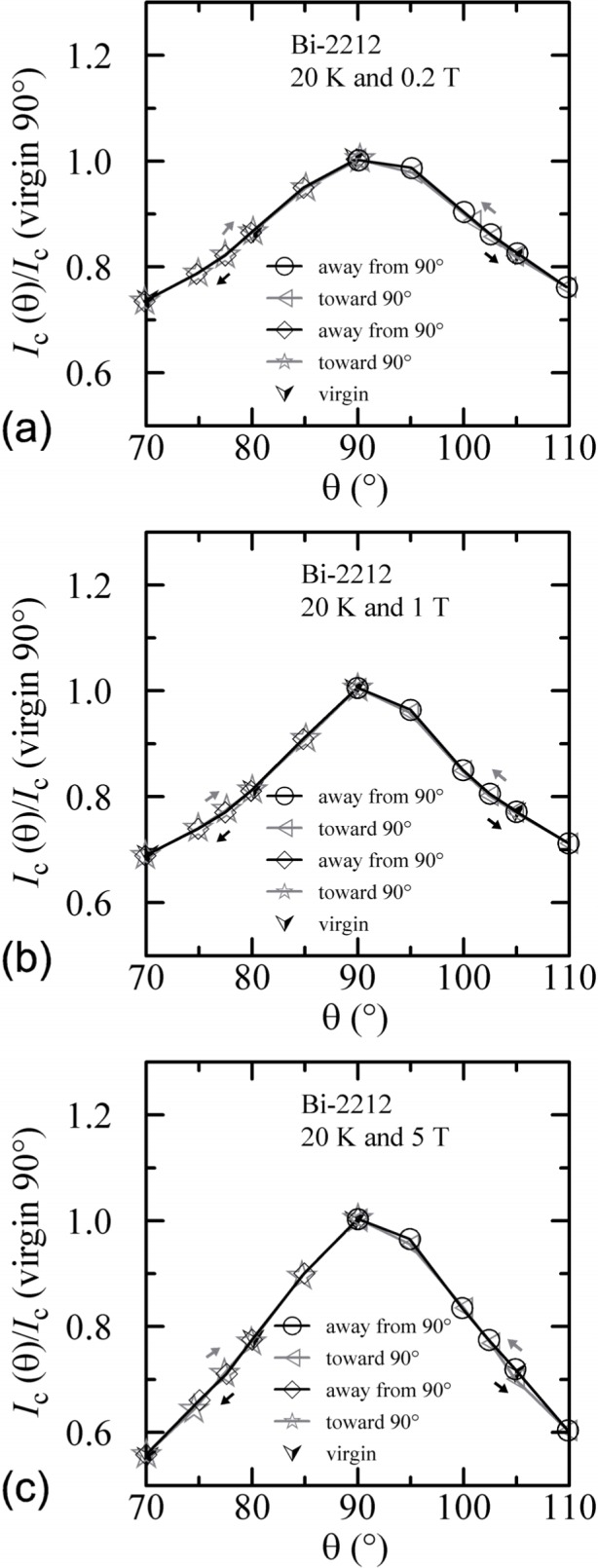
*I*_c_/*I*_c_(virgin 90°) at 0.1 µV/cm versus angle around 90° for the Bi-2212 specimen at 20 K for various sweep directions and magnetic fields: (a) 0.2 T, (b) 1 T, (c) 5 T.

**Fig. 29:**
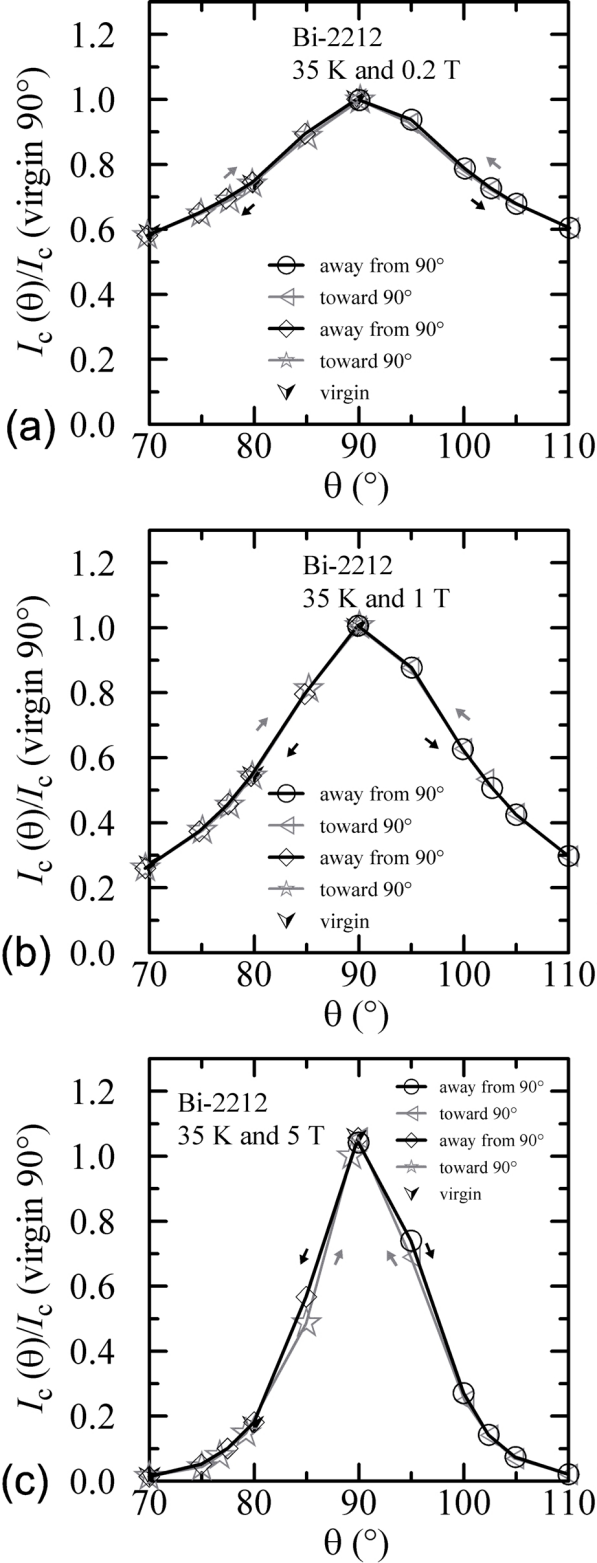
*I*_c_/*I*_c_(virgin 90°) at 0.1 µV/cm versus angle around 90° for the Bi-2212 specimen at 35 K for various sweep directions and magnetic fields: (a) 0.2 T, (b) 1 T, (c) 5 T.

An additional minor angle sweep around 90° was made after each of the previous sets of data. These minor sweeps are shown on Figs. 27–29. The branch sequence is given in the legend of each plot. For each plot, the first branch was taken going from 90° to 110°, thus continuing in the same direction as the previous data, which was from −90° to 90°. The bumps when approaching 90° from each side are nearly symmetric. As the amount of the hysteresis decreases, all branches approach the virgin values. Using the symmetry of virgin values for the 4 K and 20 K plots and the sweep branches for all of these plots, the center of symmetry seems to be between 91° and 92°. As stated earlier, this could be due to a slight error in the initial alignment of the cryostat.

**Fig. 30:**
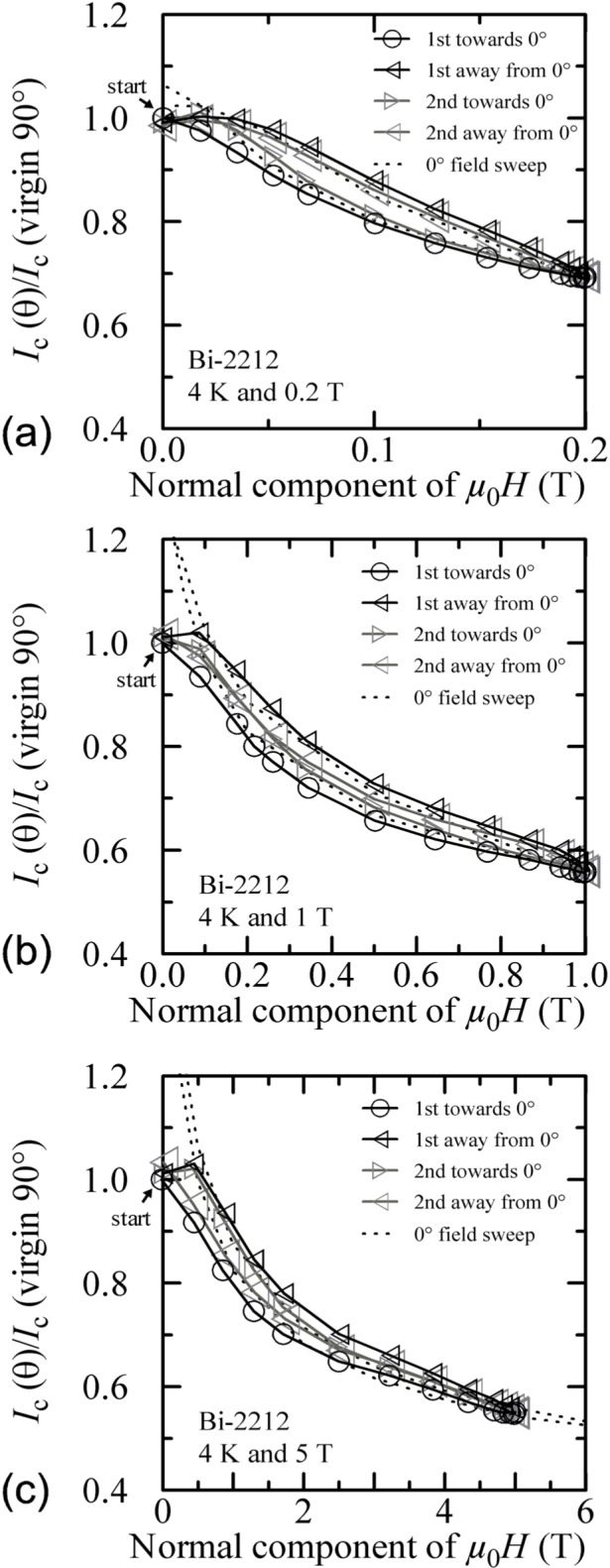
*I*_c_/*I*_c_(virgin 90°) at 0.1 µV/cm versus the normal component of the magnetic field for the Bi-2212 specimen at 4 K for the various angle sweeps and fields shown in Fig. 24: (a) 0.2 T, (b) 1 T, (c) 5 T. The dash curves show the *I*_c_ versus magnetic field curves at 0° taken from Fig. 11.

**Fig. 31:**
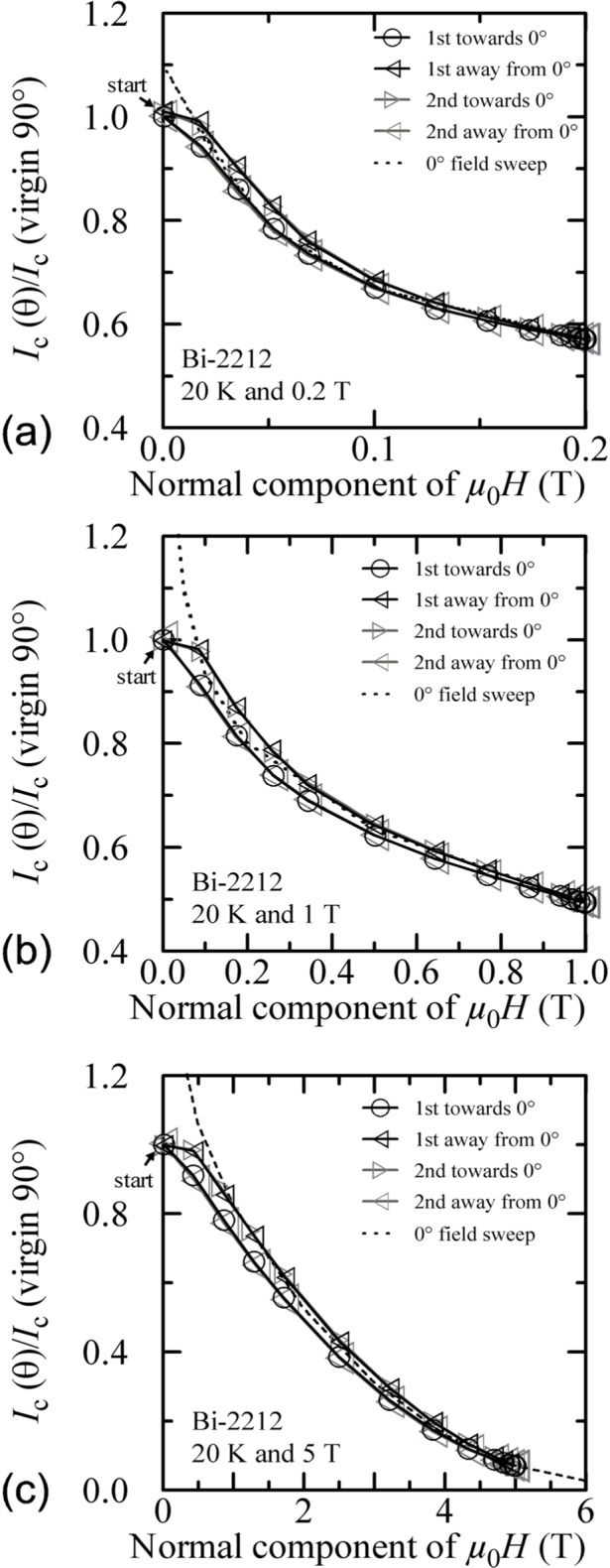
*I*_c_/*I*_c_(virgin 90°) at 0.1 µV/cm versus the normal component of the magnetic field for the Bi-2212 specimen at 20 K for the various angle sweeps and fields shown in Fig. 25: (a) 0.2 T, (b) 1 T, (c) 5 T. The dash curves show the *I*_c_ versus magnetic field curves at 0° taken from Fig. 11.

**Fig. 32:**
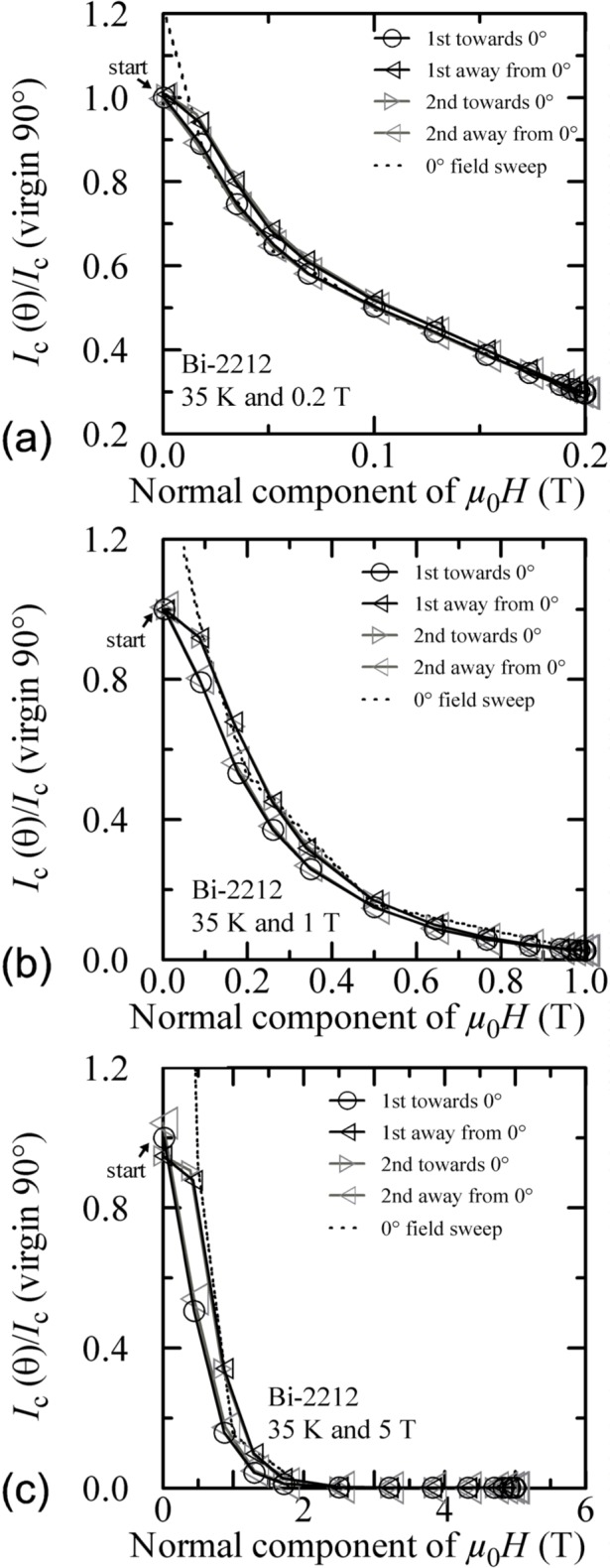
*I*_c_/*I*_c_(virgin 90°) at 0.1 µV/cm versus the normal component of the magnetic field for the Bi-2212 specimen at 35 K for the various angle sweeps and fields shown in Fig. 26: (a) 0.2 T, (b) 1 T, (c) 5 T. The dash curves show the *I*_c_ versus magnetic field curves at 0° taken from Fig. 11.

Plots of normalized *I_c_* versus the normal component of the magnetic field for all temperatures and fields are shown in Figs. 30–32. In each case, a 0**°** field-sweep to an appropriate maximum field is plotted as a dashed loop and is a good approximation to the hysteresis observed in the angle-sweep curves at a given field. These plots continue to illustrate the correlation between the amount and nature of the hysteresis observed in field-sweep and angle-sweep data.

### 3.4 Hysteresis With Temperature Sweeps

#### 3.4.1 Bi-2223

**Fig. 33:**
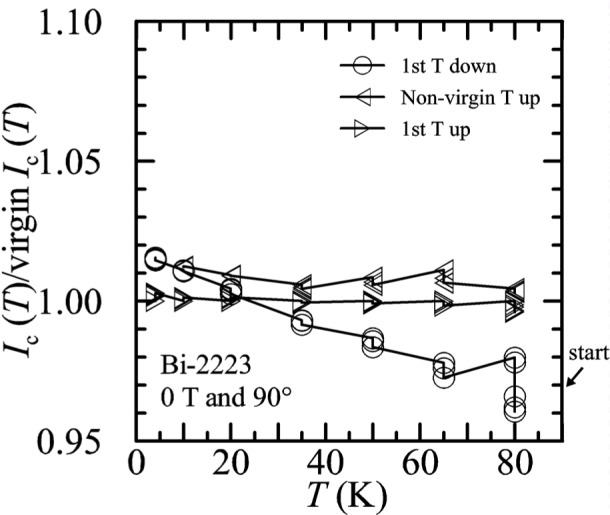
*I*_c_(T)/first *I*_c_(T) at 0.1 µV/cm versus temperature for the Bi-2223 specimen at 0 T for various sweep directions. The steps in the lines connecting the points correspond to multiple determinations of *I*_c_ at the given temperatures.

Temperature-sweep data in zero applied field for the Bi-2223 specimen are shown on Fig. 33. For each temperature, *I_c_* is normalized using the first measured value of the first-up-temperature sweep (labeled 1^st^ *T* up). For expedience and because the effect was expected to be small, individual determinations at each temperature separated by flash heating were not taken or used to normalize these data. The slight shifts between determinations at each temperature can be seen. The “1^st^ *T* down” curve was taken with decreasing temperature after a flash heating. The “non-virgin *T* up” curve is a continuation with increasing temperature after the “1^st^ *T* down” curve without a flash heating step. As expected, these zero-field curves show very little hysteresis. For temperatures of 50 K and lower, the change in *I_c_* is less than 2 % with these different sweep directions and sequence of conditions. The lack of significant hysteresis in these temperature sweeps in zero applied field indicates that the self-field generated by the specimen transport current does not significantly contribute to the hysteresis effect.

**Fig. 34:**
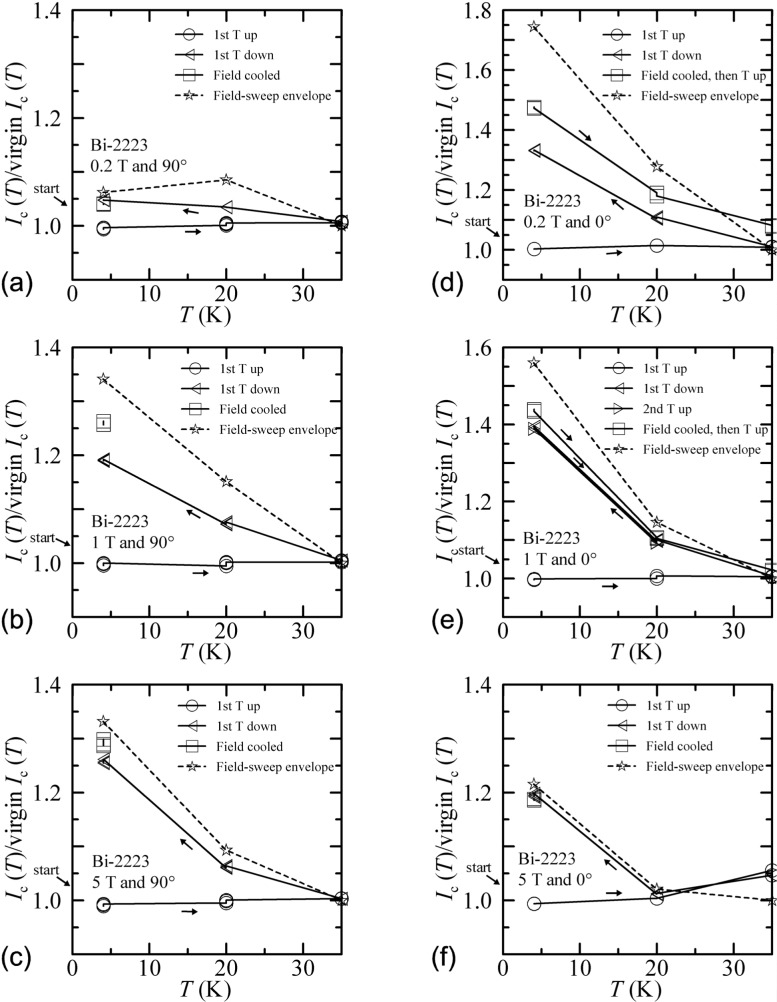
*I*_c_(T)/virgin *I*_c_(T) at 0.1 µV/cm versus temperature for the Bi-2223 specimen for various sweep directions, fields and angles: (a) 0.2 T and 90°, (b) 1 T and 90°, (c) 5 T and 90°, (d) 0.2 T and 0°, (e) 1 T and 0°, (f) 5 T and 0°. The dash curve shows the field-sweep hysteresis taken from Fig. 8, which is an envelope for the observed temperature-sweep hysteresis at each field and angle..

Figure 34 shows temperature-sweep data taken with fixed field and angle. The normalization used for these curves was slightly different from that used in the zero-field case. For the finite fields, the virgin value used to normalize these data was not taken during the temperature sweep, but was taken during separate field sweeps (with flash heating between each) at each temperature and angle. This is evident in Fig. 34f (5 T and 0°), where all of the temperature-sweep points at 35 K are different from the normalization value. Small differences at 35 K, 5 T, and 0**°** are noticeable because of the low *I_c_* values. For each of the other fields and angles, the first-up temperature sweep yields results very close to the virgin values. This is significant only at 20 K and 35 K, since the initial data at 4 K amounted to a repeat of the virgin conditions. Thus, this again is consistent with the general rule that more *correct* data are obtained by sweeping from higher *I_c_* to lower *I_c_.* The temperature-sweep hysteresis is observed as the temperature is then decreased from 35 K.

The amount of temperature-sweep hysteresis scales with that observed during the field sweeps at each corresponding temperature and angle. The dashed line in each plot in Fig. 34 reflects the hysteresis observed during field sweeps, which is also shown in Fig. 8 (plots of *I_c_(H* down)/*I*_c_(*H* up)). For example, the 4 K field-sweep envelope point in Fig. 34d is extracted from the 0**°** and 0.2 T point on Fig. 8a, which had a value of 1.74. The 20 K field-sweep envelope point in Fig. 34d is extracted from the 0**°** and 0.2 T point on Fig. 8c, which had a value of 1.28. The 35 K field-sweep envelope point has a value of 1.0 because it is at the peak temperature of the sweep. These field-sweep envelopes were obtained for field sweeps to 8 T at each temperature. The correlation between field-sweep and temperature-sweep hysteresis suggests that sweeping to higher temperatures (lower *I_c_*) is similar to sweeping to higher fields (lower *I_c_).* For the temperature sweeps where 35 K was the highest temperature, no hysteresis is expected at 35 K. There are also a few field-cooled points in these plots, where the specimen was flash heated and then cooled while the field was held constant at the value corresponding to each plot. In the field-cooled case, there can be hysteresis at 35 K. In each of the observed field-cooled results, the hysteresis was about the same or somewhat larger than observed with a temperature sweep down from 35 K. These field-cooled data were taken after the specimen returned to 4 K. In some cases (see Figs. 34d and 34e), subsequent to the 4 K measurements, the temperature was swept and measurements were made at 20 K and 35 K.

The character of the hysteresis during temperature sweeps is somewhat different from that observed with field and angle sweeps. This is likely due to the fact that the magnetic field strength and angle do not change during these temperature sweeps. As a result of this, once the sweep departs from the more *correct* branch, it never gets back to the more *correct* branch by ramping just the temperature up and down. This is shown in the “second *T* up” curve in Fig. 34e where the “second *T* up” and “first *T* down” values are nearly the same at 20 K. This is also shown in the “field cooled, then *T* up” curves in Figs. 34d and 34e where these curves at 20 K are nearly the same as or larger than the values for “first *T* down” at 20 K. In the cases of field and angle sweeps, these parameters could be swept in a manner that allows one to switch from one branch to the other, where one branch is more *correct* than the other. This cannot be done with just temperature sweeps. The initial branch can be repeated only after the field has been taken to zero, the specimen flash heated and then cooled in zero field, and the field returned to the target value.

One measurement implication of temperature-sweep hysteresis is that the temperature should be set to the target value in zero field. For example, if one wanted to measure at 20 K and 1 T, one should not start by ramping the field as one is trying to achieve temperature control at 20 K. Temperature drifts, variation, or losing temperature control while ramping the field can cause irreversible changes from the virgin branch. This was a slight factor in acquiring these temperature-sweep hysteresis data. Experimentally, it was more difficult to control the temperature undershoot during the temperature sweeps than it was to control the overshoot. The temperature overshoot was less than 0.5 K and the temperature undershoot was less than about 3 K for the 20 K point and 0 K for the 4 K point because the liquid was at 4 K. Given the direction of the temperature-sweep hysteresis, temperature overshoot is the critical factor and these overshoots were relatively minor.

The relative amount of temperature-sweep hysteresis at 0**°** and 90° changes with magnetic field. At 0.2 T, the temperature-sweep hysteresis at 0**°** is much larger than at 90°. At 1 T, the hysteresis are more similar, but higher at 0°. At 5 T, the hysteresis at 90° is a little more than at 0°. Each of these statements is also true for the field-sweep hysteresis envelopes shown in these plots in Fig. 34.

Temperature-sweep hysteresis has implications for specimen testing and could also have implications for some applications. The lack of hysteresis when raising the temperature at a constant field means that the following two paths are equivalent: (1) cooling to 4 K in zero field, increasing the field to a certain value, and then increasing the temperature to 20 K, and (2) cooling to 20 K in zero field and then increasing the field to a certain value. The observed hysteresis means that the following two paths are not equivalent: (1) cooling to 35 K in zero field, increasing the field to a certain value, and then decreasing the temperature to 20 K, and (2) cooling to 20 K in zero field and then increasing the field to a certain value. These two paths are not equivalent even though they both start and end at the same points. Can this be used to enhance the superconductor properties in a magnet application? If one could cool the magnet in an applied field of 1 T, then one would get an enhancement of about 10 % at 20 K for both 0**°** and 90°, 20 % at 4 K for 90°, and 40 % at 4 K for 0°. However, implementing this field cooling could be as difficult as taking advantage of the field-sweep and angle-sweep hysteresis. Using the same magnet to generate this field does not make sense since if one needs enhancement to operate at a given field and lower temperature, how does one expect to obtain that field at a higher temperature? The only self-contained approach would be to cool to a lower temperature (where one would have a higher *I_c_* and perhaps better cooling) and ramp the magnet to field higher than the target. Then one may obtain a higher performance at the target field and a higher temperature. Notice that this involves a combination of field-sweep and temperature-sweep hysteresis. A limiting factor is that all forms of hysteresis tend to decrease at the higher temperatures.

#### 3.4.2 Bi-2212

**Fig. 35:**
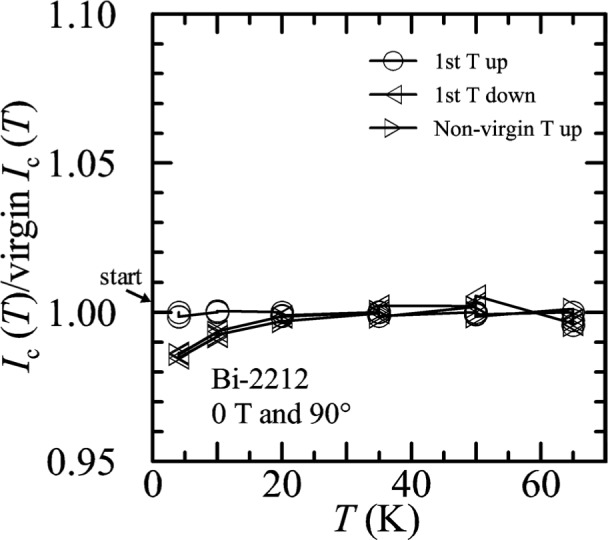
*I*_c_(T)/first *I*_c_(T) at 0.1 µV/cm versus temperature for the Bi-2212 specimen at 0 T for various sweep directions.

Temperature-sweep data in zero applied field for the Bi-2212 specimen are shown on Fig. 35. For each temperature, *I_c_* is normalized using the first measured value of the first-up-temperature sweep (labeled *1^st^ T up).* The slight shifts between determinations at each temperature can be seen. The “1^st^ *T* down” curve was taken with decreasing temperature after a flash heating. The “non-virgin *T* up” curve is a continuation with increasing temperature after the “1^st^ *T* down” curve without a flash heating step. As expected, these zero-field curves show very little hysteresis. For temperatures of 65 K and lower, the change in *I_c_* is less than 2 % with these different sweep directions and sequence of conditions.

**Fig. 36:**
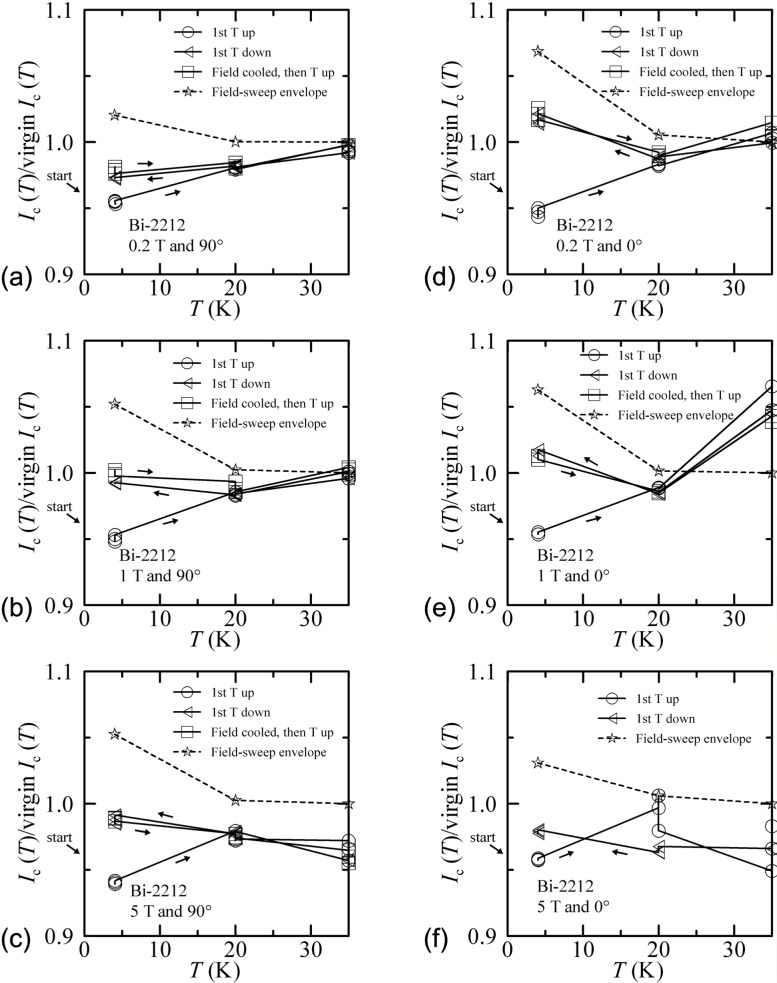
*I*_c_(T)/virgin *I*_c_(T) at 0.1 µV/cm versus temperature for the Bi-2212 specimen for various sweep directions, fields and angles: (a) 0.2 T and 90°, (b) 1 T and 90°, (c) 5 T and 90°, (d) 0.2 T and 0°, (e) 1 T and 0°, (f) 5 T and 0°. The dash curve shows the field-sweep hysteresis taken from Fig. 13, which is an envelope for the observed temperature-sweep hysteresis at each field and angle..

Figure 36 shows temperature-sweep data taken with fixed field and angle. The normalization used for these curves was slightly different from that used in the zero-field case. For the finite fields, the virgin value used to normalize these data was not taken during the temperature sweep, but was taken during separate field sweeps (with flash heating between each) at each temperature and angle. This is evident in all figures due to a slight degradation of *I_c_* with time (as shown in the Sec. 3.5.2) for this Bi-2212 specimen. For this specimen, the first sets of field- and angle-sweep data were taken at 4 K, then at 20 K and 35 K, and then the temperature sweep data were taken. Thus, the normalized values of the first-temperature-up sweep are the lowest (about 0.96) at 4 K and generally closer to 1.0 at 35 K. This causes the first-temperature-up curve to tend to have a slope, even though it is expected to be the baseline with hysteresis. The low level of hysteresis for this specimen complicates this. The amount of temperature-sweep hysteresis scales with that observed during the field sweeps at each corresponding temperature and angle. The dashed line in each figure shows the hysteresis observed during field sweeps, which gives a good envelope for this effect. The field-cooled points were not taken for Fig. 36f since the specimen was normal at 0°, 35 K, and 5 T For all of these figures, the hysteresis in the field-cooled results was nearly the same as when the temperature was decreased from 35 K.

### 3.5 Time Dependence

Both specimens, Bi-2223 and Bi-2212, exhibited a slight drift in *I_c_* with repeat determinations under some conditions. This drift was not studied extensively here however, all observations on these specimens were consistent with those reported in Ref. [7]. In this previous paper, the repeatability of *I*_c_ was studied with the specimens in different hysteresis states (virgin, enhanced, and depressed). The virgin states that were studied were at finite fields along the initial branch of a field sweep. The enhanced states were at finite fields along the descending branch of a field sweep where *I*_c_ was higher than that of the initial branch. The depressed states were at zero field after a field sweep where *I*_c_ was lower than that of the initial branch. *I*_c_ was measured throughout 800 to 1000 current cycles for each state. The multiple determinations of *I*_c_ were most consistent when the specimen was in the initial state. *I*_c_ would gradually decrease with each current cycle when the specimen was in the enhanced state. *I*_c_ would gradually increase with each current cycle when the specimen was in the depressed state. Current cycles were not effective in removing the differences from the initial state. It was faster and more complete to reduce the field to zero and flash heat the specimen to recover the virgin state of hysteresis. In certain cases, some drift in *I*_c_ can be observed in the repeat determinations shown in some plots in this present paper.

#### 3.5.1 Bi-2223

**Fig. 37:**
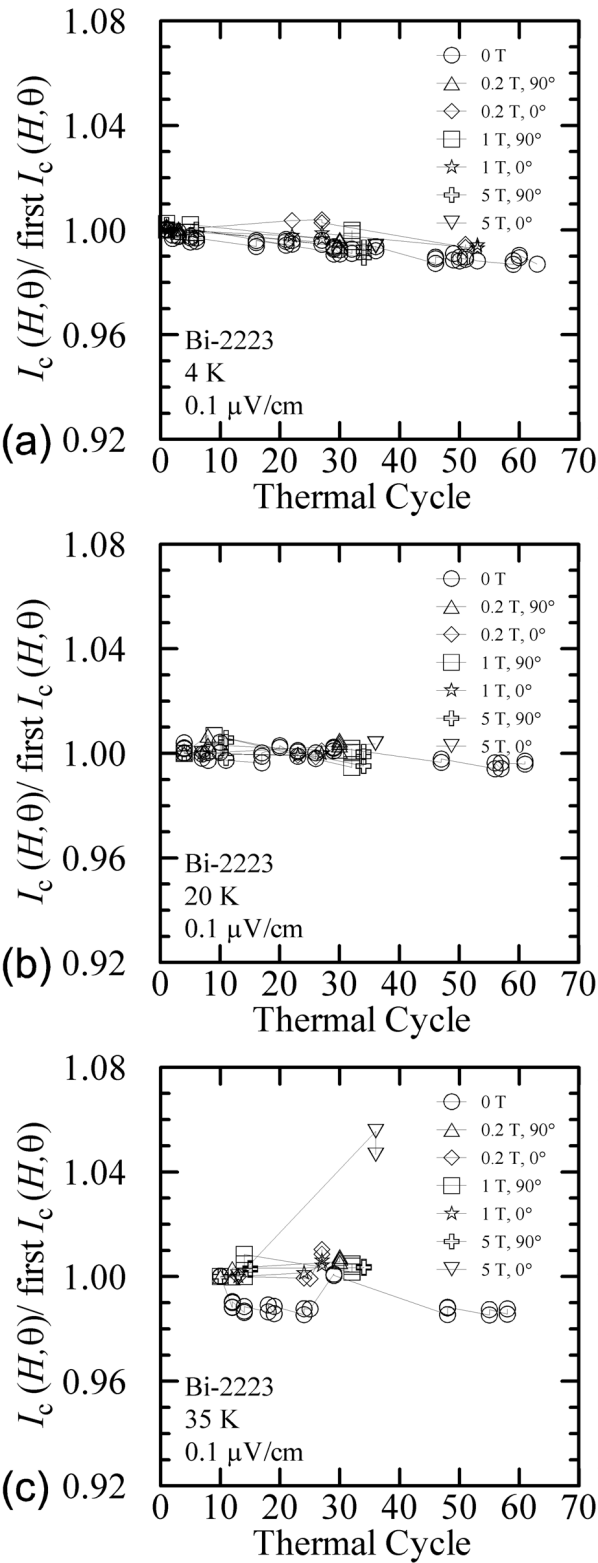
*I*_c_(*H*,)/first *I*_c_(*H*,) at 0.1 µV/cm versus time for the Bi-2223 specimen for various temperature, fields, and angles: (a) 4 K, (b) 20 K, (c) 35 K.

The number of thermal cycles and time necessary to acquire these sets of data under various conditions has the potential of degrading the properties of the specimen. The Bi-2223 specimen was thermally cycled 63 times over the 16 days that the specimen was measured. These 16 measurement days spanned a time period of about 2 months. Figure 37 shows the change in normalized *I*_c_ at various temperatures, fields, and angles versus thermal cycle number. Each *I*_c_ was determined under virgin conditions and is normalized to the first determination at each temperature, field, and angle. The plot at 4 K, Fig. 37a, is most complete since the first measurements were done at 4 K and values were often checked at 4 K and zero field before other data sets were acquired. Since the initial normalization occurred at different times, especially for the different temperatures, the slope of each line needs to be compared. The higher relative uncertainty of the lower *I*_c_’s cause more variability. Checking for degradation at various temperatures, fields, and angles has the potential to reveal degradation that appears or is more evident only under specific conditions. Within the uncertainties of these measurements it appears that the observed degradation was nearly the same for all conditions. The slope of the degradation for the Bi-2223 specimen was about 0.02 % per thermal cycle.

The residual (or remnant) magnetic field of the superconducting background magnet is not expected to noticeably affect the results presented in this paper. The maximum remnant field is about 2 mT. The Earth’s magnetic field was not screened, but its effect would be much less than that of any remnant field. Some of the measurements in Fig. 37 were made with the magnet in its virgin state where the remnant field would be zero. The effect of the remnant field is expected to be the largest at zero field. Comparing the results on Fig. 37 that had and did not have a virgin magnet does not indicate that the remnant field had a noticeable effect.

#### 3.5.2 Bi-2212

**Fig. 38:**
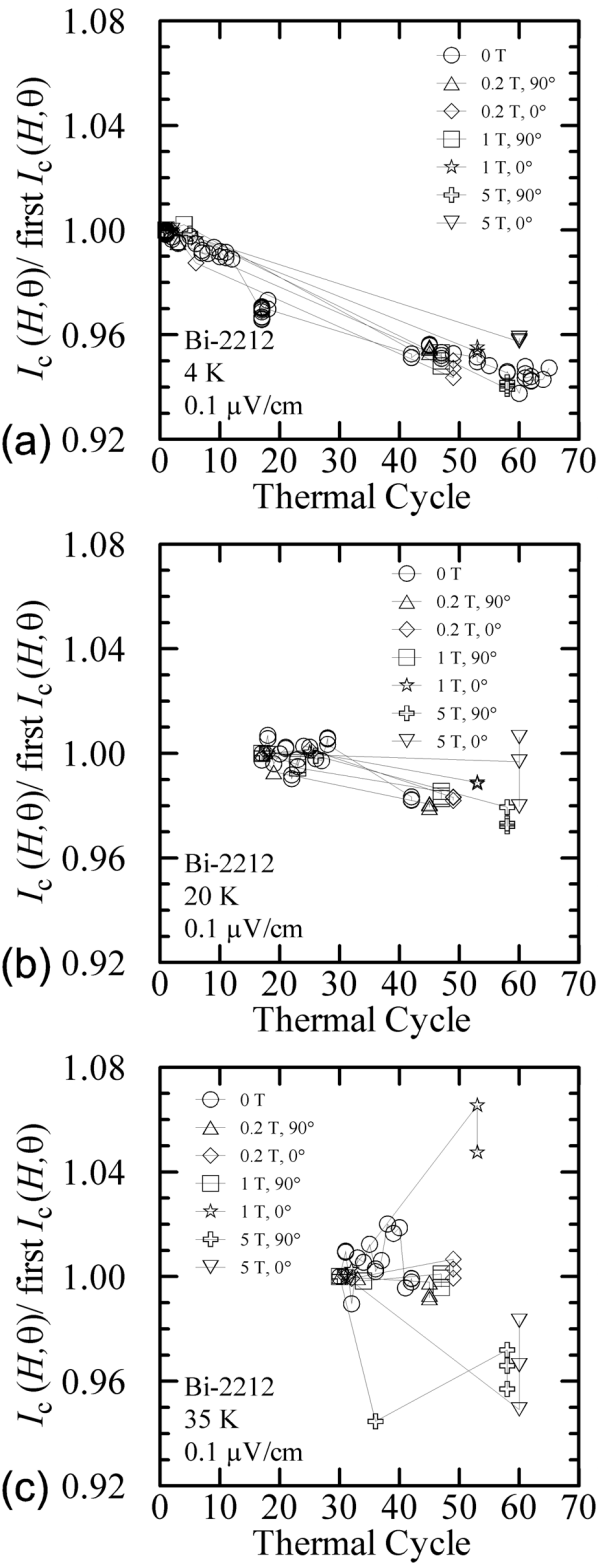
*I*_c_(*H*,)/first *I*_c_*(H*,) at 0.1 µV/cm versus time for the Bi-2212 specimen for various temperature, fields, and angles: (a) 4 K, (b) 20 K, (c) 35 K.

The Bi-2212 specimen was thermally cycled 71 times over the 18 days that the specimen was measured. These 18 measurement days spanned a time period of about 2 months. Figure 38 shows the change in normalized *I_c_* at various temperatures, fields, and angles versus thermal cycle number. Each *I_c_* was determined under virgin conditions and is normalized to the first determination at each temperature, field, and angle. Again, the plot at 4 K, Fig. 38a, is most complete. The higher variability at 20 K and 5 T and at 35 K and fields of 1 T and higher is quite evident. Within the uncertainties of these measurements it appears that the observed degradation was nearly the same for all conditions. The slope of the degradation for the Bi-2212 specimen was about 0.1 % per thermal cycle.

## 4. Conclusions

We have shown that accurate high-current variable-temperature measurements can be made. This was verified by comparing measurements made in liquid helium and in flowing helium gas at various temperatures on a commercially produced multifilamentary Cu/Nb-Ti specimen to currents over 400 A with an uncertainty equivalent to a temperature uncertainty of **±** 50 mK. Measurements can be made with different acquisition methods and current ramp rates up to 10 kA/s.

Hysteresis in the measured critical current *I_c_* was studied on two commercially produced multifilamentary HTS tape specimens: Ag-matrix (Bi,Pb)2Sr_2_Ca2Cu3O1_0–_*_x_* (Bi-2223) and AgMg-matrix Bi_2_Sr_2_CaCu_2_O_8+_*_x_* (Bi-2212). *I_c_* hysteresis was observed to cause measured values to be as much as 74 % higher or 9 % lower than the *correct* value. Which *I_c_* value is *correct* depends on the sequence of conditions in the application. Most applications cannot easily take advantage of the enhanced *I_c_* observed during specimen testing due to the nature of this hysteresis. The *I_c_* hysteresis in Bi-2223 conductors is much larger than in Bi-2212 conductors. In both cases, the size of the hysteresis effect decreases with increasing temperature.

In all, we have measured *I_c_* hysteresis on specimens from four different commercially produced multi-filamentary Bi-2223 samples. These four samples were obtained from three different manufacturers. The Bi-2223 *I_c_* hysteresis that we report here was similar in size and character to that which we observed on all of the Bi-2223 specimens. *I_c_* hysteresis results from two of these Bi-2223 specimens (other than the one Bi-2223 specimen detailed in this paper) are reported in Ref. [7].

If *I_c_* hysteresis is observed when the field is ramped up and down, then it will exist when the field angle is swept or the temperature is swept. The observed field-sweep hysteresis can be used to estimate or determine limits to the hysteresis observed in both angle and temperature sweeps. These relationships can be used to estimate how much hysteresis will be observed in different materials or different sequences of conditions. Some of the features observed in angle-sweep data are artifacts of *I_c_* hysteresis that result in an incorrect determination of the sensitivity of *I_c_* to field angle.

In general, for field, angle, and temperature sweeps, the sweep data are more *correct* if the sweep starts where the critical current is the highest and goes to where it is the lowest. Ramping the magnet to a lower or zero field and then back up to the test field can reduce the effects of *I_c_* hysteresis. These procedures can be used to reduce the effect of hysteresis and allow for approximate specimen characterization to be made in a more expedient manner.

In general, the initial and ascending branches for *I_c_* measurement are: (1) increasing magnetic field, (2) rotating toward 0**°** (bad angle), and (3) increasing temperature. The descending branches for *I_c_* measurement are: (1) decreasing magnetic field, (2) rotating toward 90° (good angle), and (3) decreasing temperature. These hysteresis data are consistent with the model that as *I_c_* is lowered along the initial branch, the magnetic field penetrates regions of the superconductor that have a higher pinning force and some flux lines remain pinned in these regions along the descending branch. Due to the interactions between all flux lines, these strongly pinned flux lines raise the measured *I_c_* along the descending branch. Without an applied field, the hysteresis effects with increasing and decreasing temperature are minimal. For temperature sweeps in finite fields, the subsequent ascending branch for a temperature sweep yields the same result as the descending branch. This is in contrast to field and angle sweeps where the subsequent ascending branch tends to approach the initial branch. This suggests that the flux lines move out of the higher pinning force sites along the descending branch of field and angle sweeps. Without a change in the field magnitude or angle, the temperature-sweep hysteresis retains the enhanced *I_c_*, which suggests that the flux lines remain in the higher pinning force regions during temperature sweeps at constant field and angle.

If significant *I_c_* hysteresis exists, the *correct* sequence of testing conditions appropriate for most HTS applications consists of magnetic field sweeps with the specimen at a fixed angle and a fixed temperature and with the specimen first heated to near its critical temperature and then cooled in zero field between magnet field sweeps at different angles or temperatures. Heating the specimen to near its critical temperature and then cooling in zero field resets the hysteresis to an initial/virgin state. A very complete characterization is needed to properly design a magnet if hysteresis is observed. It may be important to know the *I_c_* for the descending branch for ac loss or stability calculations.

Other conclusions are: (a) the lowest, repeatable *I_c_* is not always the *correct* value, (b) overshoot or drift in the field can cause significant differences in the measured *I_c_*, (c) a smooth sweep of field angles is needed to obtain reproducible results, (d) overshoot or variations in the temperature can change the measured *I_c_*, and (e) similar hysteresis was observed at criteria of 0.1 µV/cm and 1 µV/cm.

## Footnotes

1 Certain materials are identified in this paper to foster understanding. Such identification does not imply recommendation or endorsement by the National Institute of Standards and Technology, nor does it imply that the materials identified are necessarily the best available for the purpose.
